# Genomic acquisition of a capsular polysaccharide virulence cluster by non-pathogenic *Burkholderia *isolates

**DOI:** 10.1186/gb-2010-11-8-r89

**Published:** 2010-08-27

**Authors:** Bernice Meng Qi Sim, Narisara Chantratita, Wen Fong Ooi, Tannistha Nandi, Ryan Tewhey, Vanaporn Wuthiekanun, Janjira Thaipadungpanit, Sarinna Tumapa, Pramila Ariyaratne, Wing-Kin Sung, Xiao Hui Sem, Hui Hoon Chua, Kalpana Ramnarayanan, Chi Ho Lin, Yichun Liu, Edward J Feil, Mindy B Glass, Gladys Tan, Sharon J Peacock, Patrick Tan

**Affiliations:** 1 Genome Institute of Singapore, 60 Biopolis Street, 138672 Singapore; 2 Department of Microbiology and Immunology, Faculty of Tropical Medicine, Mahidol University, 420/6 Rajvithi Road, Bangkok 10400, Thailand; 3 Mahidol-Oxford Tropical Medicine Research Unit, Faculty of Tropical Medicine, Mahidol University, 420/6 Rajvithi Road, Bangkok 10400, Thailand; 4 Scripps Translational Science Institute, The Scripps Research Institute, 3344 North Torrey Pines Court, Suite 300, La Jolla, CA 92037, USA; 5 Department of Computer Science, National University of Singapore, 13 Computing Drive, 117417 Singapore; 6 National Cancer Centre Singapore, 11 Hospital Drive, 169610 Singapore; 7 Defense Medical and Environmental Research Institute, DSO National Laboratories, 27 Medical Drive, 117510 Singapore; 8 Department of Biology and Biochemistry, University of Bath, Claverton Down, Bath, BA2 7AY, UK; 9 Centers for Disease Control and Prevention, 1600 Clifton Road MS G-34, Atlanta, GA 30333, USA; 10 Department of Medicine, University of Cambridge, Addenbrooke's Hospital, Cambridge CB2 2QQ, UK; 11 Duke-NUS Graduate Medical School, 8 College Road, 169857 Singapore

## Abstract

**Background:**

*Burkholderia thailandensis *is a non-pathogenic environmental saprophyte closely related to *Burkholderia pseudomallei*, the causative agent of the often fatal animal and human disease melioidosis. To study *B. thailandensis *genomic variation, we profiled 50 isolates using a pan-genome microarray comprising genomic elements from 28 *Burkholderia *strains and species.

**Results:**

Of 39 genomic regions variably present across the *B. thailandensis *strains, 13 regions corresponded to known genomic islands, while 26 regions were novel. Variant *B. thailandensis *isolates exhibited isolated acquisition of a capsular polysaccharide biosynthesis gene cluster (*B. pseudomallei*-like capsular polysaccharide) closely resembling a similar cluster in *B. pseudomallei *that is essential for virulence in mammals; presence of this cluster was confirmed by whole genome sequencing of a representative variant strain (*B. thailandensis *E555). Both whole-genome microarray and multi-locus sequence typing analysis revealed that the variant strains formed part of a phylogenetic subgroup distinct from the ancestral *B. thailandensis *population and were associated with atypical isolation sources when compared to the majority of previously described *B. thailandensis *strains. In functional assays, *B. thailandensis *E555 exhibited several *B. pseudomallei*-like phenotypes, including colony wrinkling, resistance to human complement binding, and intracellular macrophage survival. However, in murine infection assays, *B. thailandensis *E555 did not exhibit enhanced virulence relative to other *B. thailandensis *strains, suggesting that additional factors are required to successfully colonize and infect mammals.

**Conclusions:**

The discovery of such novel variant strains demonstrates how unbiased genomic surveys of non-pathogenic isolates can reveal insights into the development and emergence of new pathogenic species.

## Background

The evolution of pathogen virulence is a complex process involving macrogenomic processes, such as large-scale gene acquisition and loss, combined with more subtle modifications of existing genes and regulatory pathways. Previous studies have shown that microbial pathogens can employ a variety of molecular factors to enable human and animal infection, such as type III toxin secretion systems, adhesins, and modulators of host signaling pathways [1-4]. As the compendium of virulence factors increases alongside the growing numbers of sequenced pathogen genomes [5], important evolutionary questions that arise include understanding how non-pathogenic species originally acquired these virulence components, investigating relationships between these virulence components to determine if their sequence of acquisition is stochastic or stereotypic, and identifying specific ecological forces in the host or environment leading to virulence gene propagation and maintenance in natural bacterial populations.

The closely related Gram-negative microbes *Burkholderia pseudomallei *(Bp) and *B. thailandensis *(Bt) represent a useful comparative system for studying the intricacies of pathogen evolution. While both species can be isolated from soil, Bp is the causative agent of melioidosis, a serious infectious disease of humans and animals with an overall fatality rate of 50% in northeast Thailand and 20% in Northern Australia [6], while Bt is generally considered non-pathogenic to mammals [7-10]. Traditional methods for distinguishing Bt from other *Burkholderia *species (including Bp) include differences in colony morphologies, arabinose assimilation, latex agglutination and immunoflourescence assays using monoclonal exopolysaccharide antibodies, along with PCR detection of arabinose or type III secretion genes and 16 s rRNA sequencing [11-22]. Previous genome comparisons have revealed several genetic differences between Bp and Bt, some of which are likely required for Bp to colonize and infect mammals [23,24]. These include the gain of a Bp-specific capsular polysaccharide gene cluster [25], the loss of an arabinose assimilation operon [26], the gain of a phosphonate utilization operon [24], the gain of a *Yersinia*-like fimbriae cluster [24], and fine scale genetic modifications in certain virulence genes, most notably those related to type III secretion [24,27]. Amongst these factors, it is unclear as to the timescale of acquisition and which are most important in precipitating mammalian virulence. Answering this question is particularly challenging due to uncertainties concerning which ecological conditions in the environment might have favored acquisition of particular virulence factors.

In this study, we hypothesized that due to its intrinsic multi-factorial nature, it is likely that virulence and non-virulence is not a black and white issue and that natural bacterial populations should contain different shades of grey corresponding to intermediate states of pathogenic potential. Moreover, we reasoned that the identification and genetic characterization of such variants, combined with relevant ecological data, should present a promising approach to addressing questions relating to the emergence of new virulent forms. To test this idea, we used a novel *Burkholderia *pan-genome array covering 28 publicly available *Burkholderia *genome sequences to profile a panel of natural Bt isolates. Remarkably, we discovered the existence of variant Bt strains exhibiting isolated acquisition of a capsular polysaccharide biosynthesis gene cluster (Bp-likeCPS) displaying features highly similar to a comparable gene cluster in Bp known to be essential for mammalian virulence [25,28-32]. Subsequent experimental, phylogenetic and genomic analyses revealed that these variant strains exhibit several functional and molecular features that are distinct from previously described Bt strains, and may represent a significant early transition towards a new virulent form. Our ability to uncover these novel variant strains demonstrates the benefit of analysis of non-pathogenic strains related to a particular pathogen of interest, and how discovery of rare variant isolates possessing subsets of virulence-related molecular features may prove useful for studying early events in pathogen evolution.

## Results

### Design and validation of a *Burkholderia *pan-genome array

We designed a *Burkholderia *pan-genome array (BPGA) containing genomic elements from 28 *Burkholderia *genomes (Additional file 1). Using a custom species-specific analysis pipeline, we identified regions of novel genetic sequence from strains belonging to each species, and concatenated these novel regions to the species reference genomes (K96423 for Bp and E264 for Bt; Additional file 2). On average, we identified 0.15 Mb of novel genetic sequence for every new Bp strain, and 0.31 Mb of novel sequence for every new Bt strain. No appreciable decrease in the rate of discovery was observed with the successive addition of strains (Additional file 1), suggesting that the Bp and Bt genomes are 'open'. The composition of the BPGA was confirmed to be independent of strain order (Additional file 3). In addition to Bp and Bt, we also incorporated genes found in *Burkholderia cenocepacia *(Bc strain J2315) [33] but not Bp K96243. The final 22.3 Mb BPGA contained genomic sequences from 23 Bp strains (12.34 Mb), 4 Bt strains (7.35 Mb), including complete genome sequences of the Bp K96243 and Bt E264 reference genomes, and 3,019 additional Bc J2315 genes (2.64 Mb). Experimental validation of the BPGA was achieved by performing a series of array-based comparative genomic hybridization (aCGH) experiments, hybridizing to the BPGA genomic DNAs from strains of known genome sequence, and considering the array results against independent *in silico *predictions (Additional files 4 and 5). These experiments confirmed the ability of the BPGA to rapidly identify genomic regions of species- and strain-specificity in a single hybridization experiment.

### Genomic variation in the *B. thailandensis *genome

**Figure 1:**
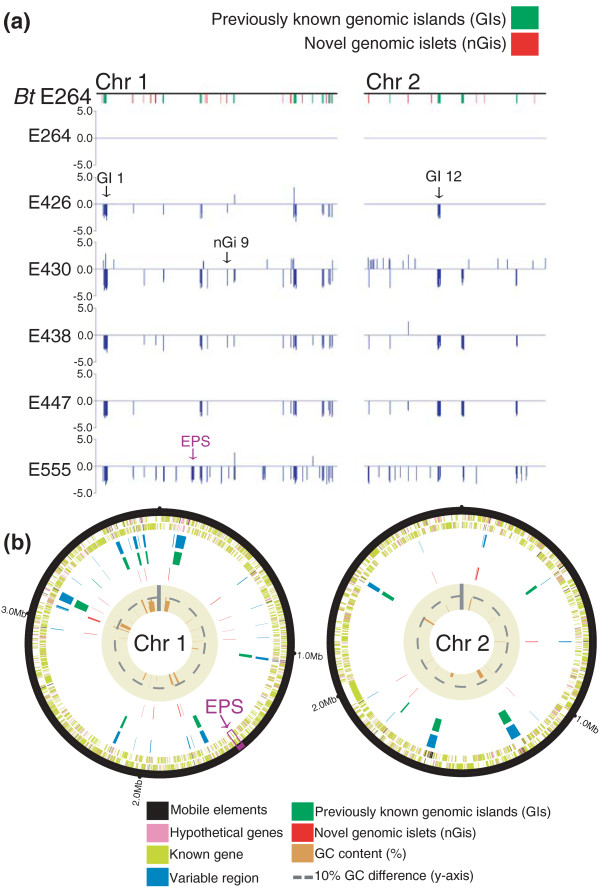
**Global identification of genomic regions of difference in Bt strains**. **(a) **BPGA hybridization patterns of natural Bt isolates compared to the BtE264 reference genome. Five Bt isolates are shown. Top row: chromosome (Chr) schematics of BtE264 Chr 1 and Chr 2. Green regions indicate previously identified genomic islands (GIs) [24], red regions indicate novel genomic islets (nGis) identified in this study. Lower rows: BGPA hybridization patterns for Bt strains. Only aCGH signals confined to the BtE264 section of the BGPA are depicted. Y-axis: log_2 _ratios of hybridization signals of each strain compared against BtE264. For all Bt strains except BtE264, regions of difference are identified by a log_2 _ratio dip or peak. Black arrows: representative GIs and nGis. The Bt exopolysaccharide (EPS) region in BtE555 is highlighted by a purple arrow. **(b) **Circular chromosomal graphs of recurrent regions of difference in Bt. aCGH results from 50 Bt isolates are depicted across BtE264 Chr 1 (left) and Chr 2 (right). Tracks are as follows, from outer to inner: first two tracks, Bt genes on forward and reverse strands (known genes (lime), mobile elements (black), hypothetical genes (pink)); blue, variable regions from aCGH data; green, previously described GIs; red, nGis identified in this study (red); brown, GC percentage of variable region compared to the Bt core genome. Grey dotted lines indicate 10% relative difference in GC content. Vertical grey line indicates the y-axis. The Bt EPS cluster is marked with a purple arrow.

Numerous studies have analyzed genomic variation in Bp [34,35], but similar genome-wide comparisons have not been reported for Bt. Using the BPGA, we performed aCGH on 50 Bt strains isolated from Thailand, Cambodia, and the USA. All isolates were confirmed to be Bt by multiple independent molecular analyses, including multi-locus sequence typing (MLST), 16 S rRNA analysis, and the presence of arabinose assimilation genes (Additional file 6). When analyzed on the BPGA, the Bt strains exhibited several regions of genomic difference compared to the BtE264 reference genome (see Figure 1a for representative strains). Many of these regions corresponded to previously identified 'genomic islands' (GIs) in BtE264 (green bars in Figure 1a), referring to chromosomal regions frequently associated with horizontal gene transfer and exhibiting unusual sequence features, such as atypical codon bias or GC content, or the presence of multiple prophage and transposon-related genes [24]. We also discovered several previously unreported smaller regions of difference across the strains, referred to as 'novel genomic islets' (nGis; red bars in Figure 1a).

Pairwise comparisons of the Bt isolates against the BtE264 reference revealed that each individual strain differed from BtE264 by approximately 3 to 5% of genomic content (mean = 3.4%), collectively representing 8% of the BtE264 genome (Additional file 7). We focused our analysis on regions exhibiting recurrent genomic variation, defined as a region exhibiting variability in at least 10% of strains. In total, we identified 39 recurrent regions of variability (Figure 1b, blue bars). Confirming previous reports that the GIs are highly mobile across different Bt strains, 13 regions corresponded to known GIs [24]. Interestingly, the exact genomic boundaries of the GIs were often found to differ slightly depending on either computational or microarray analysis. For example, the boundaries of four GIs (1, 4, 9, and 13) were discovered to be variably larger (ranging from 200 bp to 2 kb) compared to previous *in silico *analysis (Additional file 8 provides a complete list of all GIs). Because aCGH provides a direct experimental measure of regional variability in strains, these aCGH boundaries are likely more precise and thus complement and refine *in silico *analyses of bacterial genome plasticity.

Besides previously annotated GIs [24], we also discovered 26 recurrent nGis, ranging from 258 bp to 8.5 kb (median nGi size = 1.4 kb). Supporting the notion that many of these nGis are also likely to represent mobile elements, 17 of the 26 nGis exhibited several features of horizontal elements, such as insertion elements, transposases, and prophage genes, atypical GC base composition, or were located proximal to transfer RNA genes, which can function as integration hot-spots (Figure 1b) [34]. These results demonstrate that Bt genome variability between strains is not simply confined to the computationally predicted GIs, and provide a more accurate assessment of the portion of the Bt genome that is variably present across strains (the Bt accessory genome).

### Variant Bt strains exhibiting acquisition of a Bp-like capsular polysaccharide gene cluster

**Figure 2:**
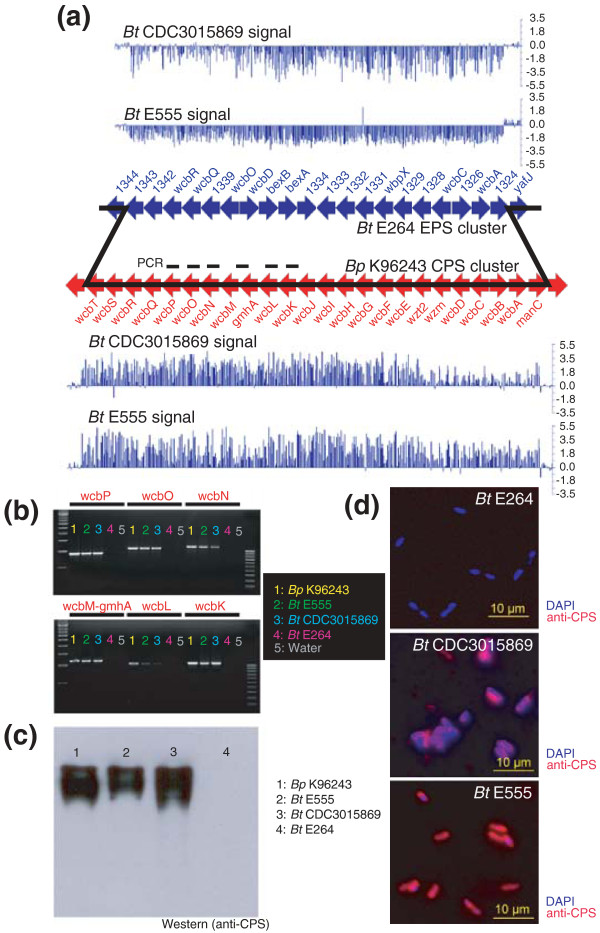
**Acquisition and expression of a Bp-likeCPS gene by variant Bt isolates**. **(a) **aCGH signals of variant Bt stains (BtCDC3015869 and BtE555) within the Bt EPS cluster region and Bp CPS cluster regions of the BPGA. Top: signal dips in the Bt EPS region (blue genes, BTH_I1324 to 1343) indicate absence of this region in both BtCDC3015869 and BtE555. Bottom: signal peaks in the Bp CPS region (red genes, *BPSL2787 *to *BPSL2810*, *wcbT-manC*) indicate gain of this region in both BtCDC3015869 and BtE555. All aCGH signals represent comparisons against BtE264. Breakpoints in both BtCDC3015869 and BtE555 are demarcated by solid black lines. **(b) **PCR detection of Bp CPS genes in variant Bt strains. Six Bp CPS genes (marked by black bars in (a) were assayed. Lanes: 1, Bp K96243 (yellow); 2, BtE555 (green); 3, BtCDC3015869 (cyan); 4, BtE264 (pink); 5, water control (grey). Also shown are 100-bp and 500-bp ladders. **(c) **Western blot analysis of BpK96243 (positive control, lane 1), and three Bt strains (BtE555, BtCDC3015869 and BtE264, lanes 2 to 4) using a monoclonal antibody directed to Bp CPS (anti-Bp CPS) demonstrates expression of cross-reacting Bp-likeCPS in BtE555 and BtCDC3015869 at around 200 kDa, but not for the negative control (BtE264, lane 4). **(d) **Immunofluorescence analysis confirms Bp-likeCPS expression in both BtCDC3015869 and BtE555, but not BtE264. Bacteria were co-stained with anti-Bp CPS antibodies (red) and DAPI (blue) to identify nuclei.

A specific region on Bt chromosome 1 (BTH_I1324-1343) encodes an exopolysaccharide (EPS) gene cluster (purple arrows in Figure 1) that in Bp has been replaced by a capsular polysaccharide gene cluster (Bp CPS) known to be essential for mammalian virulence [25,28-32]. The Bt EPS cluster was present in almost all of the profiled Bt strains; however, two isolates (BtE555 and BtCDC3015869) exhibited decreased fluorescence ratios in the Bt EPS region, suggesting absence of the Bt EPS cluster in these strains (Figures 1a and 2a). Hypothesizing that the genomic loss of Bt EPS in these strains might have been associated with the concomitant gain of novel genetic material, we surveyed hybridization signals for both BtE555 and BtCDC3015869 in the Bp-associated region of the BPGA. Both strains exhibited enhanced hybridization signals in Bp microarray probes representing Bp CPS genes (*BPSL2787-2810*) (Figure 2a), which was not observed for the other Bt strains. These results suggest that BtE555 and BtCDC3015869 may represent variant Bt strains that have acquired genomic material highly similar to Bp CPS genes. Moreover, the observation that two distinct Bt strains have gained this material suggests that this acquisition may be a recurrent event.

To validate the aCGH results, we performed a series of PCR amplification reactions using oligonucleotide primers designed against six Bp CPS genes (*BPSL2791 *to *BPSL2797*). All six PCR reactions successfully amplified PCR products from BpK96243 (positive control), BtE555 and BtCDC3015869, but not from the BtE264 reference strain (Figure 2b). Subsequent DNA sequencing of the PCR products from BtE555 and BtCDC3015869 confirmed nucleotide similarities of ≥92% to the Bp CPS genes of BpK96243 (Additional file 9). To reflect the similarity of these genes in BtE555 and BtCDC3015869 to Bp CPS, we will henceforth refer to this novel region as a 'Bp-likeCPS' cluster.

We identified the genomic boundaries of the Bt EPS/Bp-likeCPS exchange to occur at BTH_I1324 and BTH_I1343 (Figure 2a). To explore the evolutionary sequence of this genomic replacement event, we benchmarked patterns of nucleotide composition within the Bp-likeCPS cluster against the general Bt chromosome as a background model. Genes in the Bp-likeCPS cluster exhibited a markedly lower G+C content (59.2%) compared to the G+C content of the general Bt chromosome (67.3% in Bt chromosome 1 versus 65.5% in the Bt EPS), consistent with the Bp-likeCPS cluster being a foreign element recently acquired through horizontal gene transfer (Additional file 10). In contrast, genes in the Bt EPS cluster (BTH_I1328 to BTH_I1337), found in the majority of Bt strains, exhibited an average G+C highly similar to the general Bt chromosome, suggesting that this represents the ancestral genomic state. These results suggest that the variant Bt strains have acquired the Bp-likeCPS gene cluster, and that isolates containing Bt EPS, constituting the majority of previously described Bt strains, likely represent an ancestor population.

### Functional expression of Bp-likeCPS in variant Bt strains

To assess if the Bp-likeCPS cluster might be functionally expressed in the variant Bt strains, we performed immunoblotting analysis on a panel of Bp and Bt protein extracts using anti-Bp CPS antibodies [36]. Immunoreactive bands of approximately 200 kDa corresponding to Bp CPS were observed in BpK96243 positive control strains, BtE555 and BtCDC3015869 (the two variant strains), but not in BtE264 negative controls (Figure 2c). Expression of Bp-likeCPS at the surface of BtE555 and BtCDC3015869 bacteria was further confirmed by immunofluorescence imaging (Figure 2d). These data indicate that the Bp-likeCPS cluster is expressed in the variant Bt strains, and with sufficient conformational similarity to cross-react with antibodies directed towards Bp CPS.

### Genome sequencing of BtE555 confirms the presence of the Bp-likeCPS cluster

**Figure 3:**
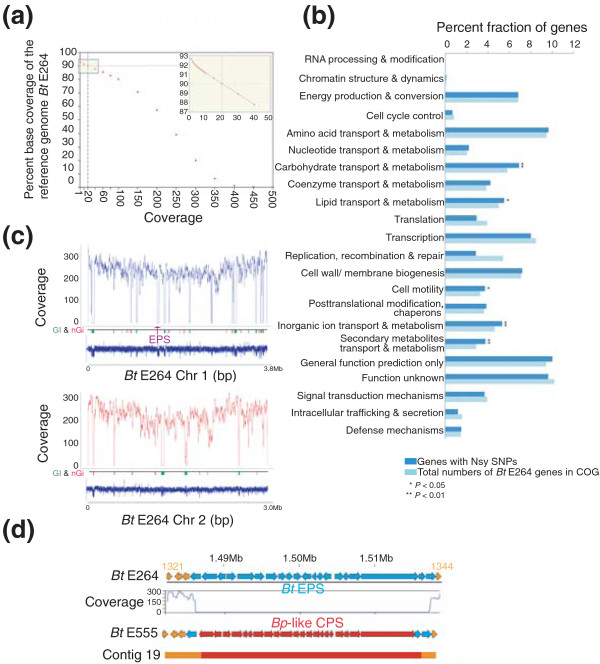
**Genome sequencing of the BtE555 variant strain**. **(a) **Reference genome coverage. BtE555 short reads were aligned against the BtE264 reference. Y-axis: percentage nucleotide coverage of the BtE264 genome. X-axis: number of reads covering each nucleotide. Vertical blue line indicates 20× coverage, corresponding to 90% coverage of the BtE264 genome. The inset graph is a close-up of the intersect. **(b) **Functional enrichment of Bt genes with nonsynonymous SNPs (Nsy SNPs). COG functional categories are indicated on the y-axis, and the percentage of genes in each COG category is shown on the x-axis. Dark blue columns represent Bt genes with Nsy SNPs relative to BtE264, and light blue columns indicate all Bt genes with COG annotations. COG categories exhibiting a significant enrichment of Bt genes with Nsy SNPs are highlighted by asterisks (*P < 0.05 or **P < 0.01, binomial test; after Bonferroni correction). **(c) **Sequence coverage gaps in BtE264 are associated with GIs and nGis. Shown are coverage maps of BtE264 chromosome 1 (top) and chromosome 2 (bottom). Graph: median fold-coverage of BtE555 sequence reads using a 10-kb moving window. Genomic track: genomic locations of predicted GIs and nGsi in green and red bars, respectively. aCGH track: aCGH hybridization patterns of BtE555 against BtE264. The x-axis shows the BtE264 genome co-ordinates (bp). **(d) **Confirmation of the Bp-likeCPS gene cluster in BtE555. Row 1: genomic coordinates of the BtE264 EPS cluster (chromosome I BTH_I1321 to BTH1344). Bt EPS cluster genes are shown in blue. Row 2: coverage map shows a drop of BtE555 sequence reads corresponding to Bt EPS genes. Row 3: genomic locations of predicted BtE555 genes in contig 19, showing genes conserved with BtE264 (orange) and Bp-likeCPS cluster genes (red). Row 4: genomic location of BtE555 contig 19 (34 kb) showing conserved (orange) and BtE555-specific regions (red).

The discovery of these variant Bt strains motivated us to perform whole-genome sequencing for a representative isolate (BtE555). Using paired-end deep sequencing (see Materials and methods), we generated >2.5 Gb of mappable BtE555 genomic sequence. Mapping of the BtE555 reads to the BtE264 reference genome revealed that approximately 90% of the BtE264 genome was covered by a minimum of 20 independent reads (that is, 20× coverage, our threshold for calling SNPs, with an overall mean genomic coverage of 170× (Figure 3a). Comparing regions conserved between both strains, we identified >29,000 SNPs between the BtE555 and BtE264 genomes. Targeted Sanger re-sequencing of 50 randomly selected SNPs yielded a false positive rate of <4%, confirming the accuracy of the identified SNPs (Additional file 11). Approximately 80% of the SNPs (23,767 SNPs) occurred in genes, yielding an overall intragenic nucleotide diversity between BtE264 and BtE555 of approximately 4.0 SNP per kilobase. Interestingly, this value is elevated relative to the average intragenic nucleotide diversity between different Bp strains (approximately 2.0 SNPs per kilobase) [37]. Close to two-thirds of the intragenic SNPs were synonymous, consistent with an overall strong selection pressure to globally conserve protein sequence and function (*P *< 2.2 × 10^-16^, binomial test) [38]. When mapped across various functional pathways (COG categories), genes containing non-synomynous SNPs (SNPs predicted to alter protein sequence) were found to be significantly enriched in pathways related to carbohydrate metabolism, inorganic ion transport, and secondary metabolism (*P *< 0.01 relative to all Bt genes, after multiple hypothesis Bonferroni correction) (Figure 3b).

The majority (64%) of BtE264 genomic regions exhibiting low coverage (< 20×) of BtE555 sequence reads were associated with variable genomic elements such as GIs, nGis, or regions with repetitive sequence, which can pose difficulties in mapping (Figure 3c). For example, we observed low coverage of the Bt EPS cluster region in BtE264, confirming the absence of this region in the BtE555 genome (Figure 3c). To identify genetic elements specifically present in BtE555 but not BtE264, we then performed *de novo *assembly of the BtE555 sequence reads and assembled 521 sequence contigs with a median (N50) size of approximately 20 kb (Additional file 12). Filtering of these contigs against the BtE264 genome identified approximately 300 kb of BtE555-specific sequence, and several of the *de novo *assembled contigs could be specifically assigned to particular low coverage regions in BtE264, such as GIs and nGis (approximately 51% of all low coverage regions; Additional file 13). For example, contig 19 (34 kb) was found to map across the Bt EPS cluster and to contain the Bp-likeCPS cluster (Figure 3d). A comparison of the Bp-likeCPS cluster against the BpK96243 CPS cluster (*BPSL2787-BPSL2810*) revealed an overall nucleotide similarity of 94.4% (96% protein similarity). Besides the Bp-likeCPS cluster, other notable genes found in BtE555 but not in BtE264 included a taurine metabolism gene cluster, and various lipocalins, which are also found in other Gram-negative bacteria like *Vibrio cholera *(Additional file 14).

### Variant Bt strains expressing Bp-likeCPS behave as part of a distinct phylogenetic subgroup

**Figure 4:**
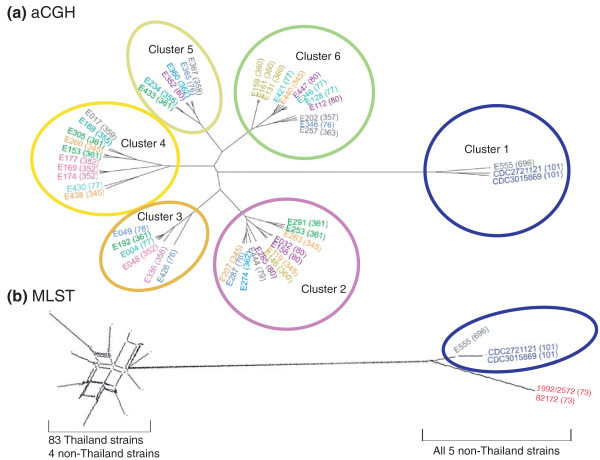
**Phylogenetic analysis based on aCGH and MLST**. **(a) **aCGH clustering of Bt isolates. Clusters were based on whole-genome microarray data. Six distinct clusters are observed, with cluster 1 containing both Bp-likeCPS-expressing strains. Strains are color-coded by the sequence types (STs), also shown in brackets. **(b) **MLST network reconstruction of Bt genotypes. MLST clustering was performed on 21 STs, 18 of which are shared with the aCGH data. 18 genotypes are represented by a highly reticulate network at the left of the figure, while three genotypes (ST73, ST101 and ST696) segregate as a distinct phylogenetic clade.

To define patterns of genetic relatedness between the variant Bt strains and the general Bt population, we performed phylogenetic analyses where relationships between the strains were inferred either by whole-genome aCGH data or MLST. Using unsupervised clustering, we grouped the Bt strains to one another based on their overall patterns of genomic similarity computed from the aCGH data, and obtained an unrooted aCGH tree with six distinct clusters (Figure 4a). The robustness of the aCGH clusters was confirmed by perturbing the microarray data and computing kappa coefficients for each cluster (see Materials and methods). A significant degree of consistency between the original aCGH clusters and clusters obtained after data perturbation was obtained (κ for all clusters ranged from 0.94 to 1, *P *< 0.01; Additional file 15). Notably, while five of the six clusters were roughly equidistant to one another in terms of relatedness, one cluster (cluster 1) behaved as a distant outlier group. Cluster 1 contains three Bt strains, of which two are Bp-likeCPS-expressing isolates (BtE555, BtCDC3015869), and BtCDC2721121. Besides Bt EPS, other genes absent in all three cluster 1 strains compared to BtE264 included various recombination related genes, permeases, and DNA binding proteins (Additional file 16). These aCGH clustering findings suggest that the variant Bt strains appear to form part of a distinct phylogenetic subgroup that is separate from other Bt strains.

To further support the whole-genome phylogeny, we repeated the phylogenetic analysis using MLST data based on the concatenated sequences of seven housekeeping loci [39]. In addition to the 17 sequence types (STs) associated with the 50 strains profiled by aCGH, we also included MLST data for an additional 42 Bt strains (Additional file 17). Collectively, the 92 Bt strains were resolved into 21 STs. Comparisons of phylogenetic trees generated using aCGH or MLST data revealed an overall concordance of 47%, with strain pairs displaying high aCGH similarities also exhibiting low MLST locus variance rates (Additional file 18). These results are consistent with previous studies demonstrating a significant degree of consistency between strain clusters established by aCGH and MLST [35,40,41]. Network reconstruction of the 21 ST genotypes revealed that a group of five Bt isolates, including the two variant Bp-likeCPS-expressing Bt strains (BtE555 (ST696), BtCDC3015869 (ST101)) and three other strains (BtCDC2721121 (ST101), Bt1992/2572 (ST73) and Bt82172 (ST73)), segregated as a phylogenetic clade distinct from a second clade containing the remaining Bt isolates, including all 83 isolates characterized from Thailand to date (Figure 4b). Similar results were obtained in a rooted tree analysis using a Bp ST (ST70) as an out-group (Additional file 19). Thus, using both aCGH and MLST data, there appears to exist a distinct subgroup of Bt isolates (STs 696, 101 and 73) that are genetically distinct from the majority of previously described Bt isolates, and this subgroup contains both Bp-likeCPS-expressing strains.

Correlation of the strain clusters to ecologic data revealed that a common theme unifying the Bt strains in the outlier subgroup was that they were all isolated from atypical geographical and isolation sources, compared to the majority of Bt strains, which were recovered in Thailand and Australia. Specifically, BtE555 was isolated in Cambodia, BtCDC3015869 and BtCDC2721121 in the USA, Bt1992/2572 in Kenya, and Bt82172 in France. In addition to these geographic differences, three of the outlier Bt strains were isolated from not soil, the typical Bt reservoir, but from mammalian hosts - BtCDC3015869 and BtCDC2721121 were isolated from human patients associated with clinical infection [42], and Bt82172 was isolated from the intestines of a horse (see Discussion). These observations suggest that Bt strains in the outlier subgroup may have an altered ability to survive in different environments and may occupy separate niches compared to previously described Bt strains.

### Bp-likeCPS-expressing Bt strains exhibit several Bp-associated phenotypes

**Figure 5:**
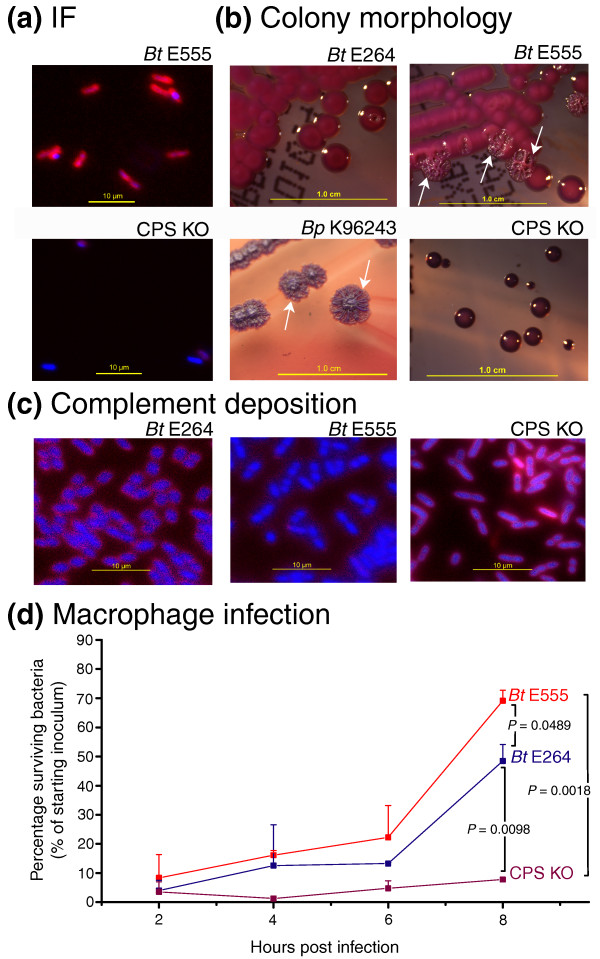
**Bp-likeCPS confers multiple Bp-associated phenotypes to BtE555**. **(a) **Immunofluorescence (IF) analysis of BtE555 and an isogenic strain disrupted in the Bp-liksCPS region (CPS KO). Strains were stained with DAPI (to identify nuclei) and the anti-Bp CPS monoclonal antibody. Wild-type BtE555 exhibits a clear distinctive halo of Bp-likeCPS expression around nuclei, while CPS KO strains exhibit either severely attenuated or absent Bp-likeCPS expression. **(b) **Colony morphologies of strains on ashdown media. BtE264 colonies (top left) are relatively round, with smooth contours, convex and glossy. In contrast, Bp K96243 colonies (bottom left) exhibit a wrinkled colony phenotype (white arrows). The BtE555 strain exhibits a mixture of smooth and wrinkled colonies (top right, white arrows), and the Bp-likeCPS KO strain (bottom right) develops small round violet colonies with no wrinkling. All strains were assayed after incubation at 37°C for 5 days. **(c) **Complement deposition assay. Strains were assayed for their ability to avoid human complement C3b deposition on cell surfaces (red staining; see Materials and methods). BtE264 is associated with abundant C3b deposition, while BtE555 exhibits minimal C3b accumulation. However, C3b deposition is clearly observed in the Bp-likeCPS KO strain. **(d) **Macrophage survival assay. Strains were assayed for their ability to survive and replicate in RAW macrophages, from 2 h to 8 h post-infection. BtE555 exhibits a highly significant ability to survive and replicate in macrophages compared to the Bp-likeCPS KO strain (*P *= 0.002). BtE555 also exhibits a statistically significant enhancement for survival and replication compared to BtE264 (*P *= 0.049).

The isolated gain of the Bp-likeCPS cluster in BtE555 provided us with the opportunity to test the sufficiency of Bp-likeCPS in contributing to various Bp-associated phenotypes. To facilitate comparisons in an isogenic strain background, we created genetic mutants of BtE555 CPS knockout (KO) where the Bp-likeCPS cluster was disrupted by the insertional mutagenesis of a targeting vector into the *wcbB *gene (see Materials and methods). Immunofluorescence studies confirmed that the CPS KO exhibited severely attenuated to absent Bp-likeCPS expression (Figure 5a). In growth experiments, the CPS KO strains exhibited slightly slower *in vitro *growth rates than BtE555, although these differences were marginal (Additional file 20).

Our previous survey of Bp isolates has shown that the predominant colony morphology (morphotype) of Bp is 'wrinkled', with wrinkled colonies being observed for approximately 75% of naturally occurring isolates [43]. In contrast, Bt colonies are typically round and smooth (Figure 5b). When cultured on Ashdown medium, BtE555 colonies exhibited a mixture of 'smooth' and 'wrinkled' colony morphologies, while CPS KO colonies developed as round colonies with no wrinkling (Figure 5b). This result suggests that Bp-likeCPS expression may contribute to the 'wrinkled' colony morphotypes of the Bt variant strains, a phenotype similar to that observed in the majority of Bp strains.

We then considered two mechanisms by which Bp-likeCPS might contribute to virulence - host immune evasion and intracellular survival. First, Bp-likeCPS might enhance virulence in organisms with an active immune system by blocking deposition of host immune factors such as complement on pathogen surfaces, thereby inhibiting detection by innate immunity pathways. In a complement deposition assay, BtE264 incubated with human serum exhibited significant binding of C3b complement on microbial surfaces (Figure 5c), indicating that Bt EPS is not capable of blocking complement deposition. In contrast, no C3b binding was observed in wild-type BtE555, while CPS KO strains exhibited C3b binding comparable to BtE264 (Figure 5c). These results suggest that Bp-likeCPS expression in the variant strain is sufficient to block binding of host complement proteins to microbial surfaces, thereby linking Bp-likeCPS to a possible virulence trait.

Second, Bp-likeCPS may also contribute to virulence by facilitating intracellular survival and replication. Using macrophage infection assays, we tested the ability of BtE555 cells to survive in an intracellular host environment. Wild-type BtE555 exhibited an almost nine-fold increase in their ability to survive and replicate in RAW macrophage cells compared to isogenic CPS KO strains (*P *= 0.002), indicating that the Bp-likeCPS is required for survival in an intracellular environment (Figure 5d). BtE555 also exhibited a significantly enhanced ability to survive in RAW macrophages compared to BtE264 (*P *= 0.049). It is worth noting, however, that the absolute difference between BtE555 and BtE264 is surprisingly modest.

### Bp-likeCPS acquisition is not sufficient to enhance virulence relative to other Bt strains

To directly test the *in vivo *virulence potential of BtE555, we conducted animal infection experiments. We used two established models: a murine intra-nasal assay and a *Caenorhabditis elegans *co-culture assay [44,45]. We infected BALB/c mice, which are highly sensitive to Bp infection, to increasing intranasal doses of Bp (BpK96243 or Bp22) and Bt (BtE264 or BtE555) and monitored the infected mice up to 13 days post-infection (Table 1). The Bp strains were highly virulent, and the majority of mice died within 13 days of receiving initial Bp inoculums ranging from 100 to 500 colony forming units (cfu; LD50 values 225 cfu and 16 cfu for Bp K96243 and Bp 22, calculated on day 13). In contrast, almost every mouse recovered after either BtE264 or BtE555 infection, and the few deaths observed only occurred when a massive inoculum was administered (> 10^6 ^cfu). Surviving BtE555-infected mice sacrificed at day 13 exhibited no visible abscess formation in the lungs, spleens, and liver, and no visible growth was observed when undiluted homogenates from lung and spleen were cultured on recovery plates. These data point to an early clearance of the initial BtE555 infection. In the *C. elegans *model, BtE555 also exhibited low levels of virulence in the worm infection assay, with killing rates that are 2 days slower than BtE264, with only 100% death after 120 h. When tested, CPS KO strains killed *C. elegans *at a slower rate than parental BtE555 strains, indicating that Bp-likeCPS may also be required for efficient nematode killing (Table 2). These results indicate that Bp-likeCPS acquisition in the variant Bt strains alone is not sufficient to enhance virulence compared to other Bt strains as assessed in an *in vivo *infection model, indicating a requirement for additional virulence modifications to colonize and infect mammals.

**Table 1:** Strain virulence in the mouse infection model (BALB/c)

Strain	Inoculum (cfu)	Mortality 4 days post infection	Mortality 13 days post infection
PBS	0	0/6	0/6
			
BpK96243	9	0/6	0/6
	118	0/6	4/6 (66.7%)
	380	0/6	4/6 (66.7%)
	1180	0/6	5/6 (83%)
	3600	1/6 (16.7%)	6/6 (100%)
			
Bp22	6	0/6	2/6 (33%)
	19	0/6	5/6 (83%)
	58	0/6	5/6 (83%)
	175	1/6 (16.7%)	6/6 (100%)
	525	2/6 (33%)	6/6 (100%)
			
BtE264	1000	0/6	0/6
	10^4^	0/6	0/6
	10^5^	0/6	0/6
	10^6^	0/6	0/6
	10^7^	1/6 (16.7%)	1/6 (16.7%)
			
BtE555	1,000	0/6	0/6
	10^4^	0/6	0/6
	10^5^	0/6	0/6
	10^6^	0/6	0/6
	10^7^	4/6 (66.7%)	4/6 (66.7)

Infection assays in BALB/c mice. Mice were monitored for up to 13 days post-infection with different starting amounts of bacteria (cfu column). Note the differences in cfu between Bp and Bt strains (starting cfu 6 to 9 versus 1,000). PBS, phosphate-buffered saline.

**Table 2:** Strain virulence in the *C. elegans *infection model

	Lethality			
	
Strain	> 24 h	> 48 h	> 72 h	> 96 h
*E. coli *OP50	0/40 (0%)	0/40 (0%)	0/40 (0%)	0/40 (0%)
BtE264	4/39 ± 3 (5%)	31/39 ± 3 (80%)	36/39 ± 3 (92%)	39/39 (100%)
BtE555	2/41 ± 2 (4%)	10/39 ± 1 (27%)	36/39 ± 1 (93%)	39/39 (100%)
CPS KO	1/37 ± 1 (0%)	3/37 ± 2 (9%)	20/37 ± 5 (53%)	35/37 ± 2 (95%)

Infection assays in *C. elegans*. Strains were monitored up to 5 days (120 h) post-infection. Death rates are shown both as raw counts and as percentages.

## Discussion

In this study, we discovered variant Bt strains exhibiting acquisition of a capsular polysaccharide gene cluster (Bp-likeCPS) bearing striking similarities to a comparable cluster in Bp that is generally regarded as an essential mammalian virulence factor [25]. Analysis of the Bp-likeCPS genes revealed that they are highly distinct from preexisting polysaccharide synthesis genes normally found in Bt. Subtractive hybridization studies and genomic comparisons have shown that that the majority of the Bp CPS gene cluster is absent in Bt [24,25,46], and the few polysaccharide-related genes in Bt exhibiting similarity to genes in the Bp CPS cluster (BTH_I1343-1338 and BTH_I1327-1324) bear a relatively low similarity score of approximately 75% (74.8% and 72.8% nucleotide and protein identity) to their Bp homologs *wcbT-wcbO *and *wcbC-manC*. More importantly, the preexisting Bt genes are also by themselves not sufficient to produce a capsular polysaccharide with functional properties of Bp CPS (for example, evasion of human complement binding, antibody cross-reactivity). In contrast, the 34-kb Bp-likeCPS cluster acquired by the variant Bt strains displays a general structure and organization matching the intact Bp CPS cluster (*wcbT-manC*), and very high similarity scores to Bp CPS genes (94.4% and 96% nucleotide and protein similarity). A representative Bp-likeCPS-expressing Bt strain (BtE555) also exhibited several Bp-like virulence traits (macrophage survival, resistance to complement deposition) in a Bp-likeCPS-dependent manner, demonstrating that the Bp-likeCPS is functionally expressed. To our knowledge, these variant Bt strains thus represent a novel subclass of strains that have not been described previously in the literature.

Phylogenetic analysis using both aCGH and MLST data revealed that the variant Bt strains occupy part of an evolutionary clade that is genetically distinct from the majority of Bt strains. Natural Bt populations from Thailand are known to exhibit an MLST population structure similar to that previously described for Bp [47-49], where large numbers of closely related STs (allelic combinations) appear to have been generated through high levels of homologous recombination between genetically conserved alleles. Supporting this, the allele profiles of the 18 ST genotypes from Thai strains are at linkage equilibrium (I_A_^s ^= -0.0475; calculated using LIAN 3.0 [50]). Within the freely recombining Bt group, the reconstruction of robust relationships is not possible by standard phylogenetic methods, and thus the relatedness between these strains is more appropriately represented as a network (Figure 4b). In contrast, the three genotypes in the outlier evolutionary subgroup represent a monophyletic cluster clearly distinct from this freely recombining group, a separation that is further supported by whole-genome aCGH data. Such phylogenetic divergence in the face of high recombination frequencies suggests that barriers to gene flow may have emerged between strains in this outlier subgroup and the regular Bt population, which could be either ecological or geographical (allopatry). For example, all three genotypes in the outlier subgroup were associated with strains external to Thailand and Australia. These findings are consistent with the possibility that we may be witnessing an incipient speciation event leading to irreversible divergence between these two groups.

Discovery of the variant Bt strains suggests that Bp-likeCPS acquisition may occur relatively freely in the natural environment, but raises questions regarding the ecological conditions under which Bp-likeCPS acquisition might have conferred a selective advantage. In the literature, Bp CPS has been previously studied primarily with respect to its role in facilitating mammalian infection by Bp, by facilitating bacterial attachment to pharyngeal epithelial cells [51] and evading complement binding [31]. Bp CPS mutants have been shown to exhibit severely attenuated levels of virulence in multiple mammalian animal models [25,28,29], and anti-Bp CPS antibodies can also protect mice against extremely high dosages of Bp [32]. However, BtE555 did not exhibit enhanced levels of virulence relative to other Bt strains in a highly sensitive animal model of acute melioidosis, and recently, BtCDC3015869, another Bp-likeCPS-expressing variant strain, has also been shown to exhibit low levels of virulence in hamsters and mice [10]. The low levels of mammalian virulence associated with the variant Bt strains, which are similar to regular Bt strains, leaves open the possibility that the major ecological force driving Bp-likeCPS acquisition in these variant strains might not have been a specific requirement to infect mammals. For example, as environmental microorganisms, Bt and Bp must have developed mechanisms to survive environmental stresses encountered in soil, such as desiccation, rapid changes in temperature, pH, water content, and attack by other bacteria, nematodes and amoebae, and it is tempting to speculate a need to achieve improved environmental fitness might have provided the true evolutionary force for Bp-likeCPS acquisition. Under this concept, understanding the 'function' of these genes, as viewed by natural selection, may thus require consideration of the evolutionary forces acting within the soil ecosystem. Probing this issue represents an interesting area for future research.

Finally, beyond their distinct geographical localities, ecologic data also suggest that Bt strains in the outlier subgroup (including the Bp-likeCPS expression strains), may have a subtly altered ability to survive in different environments and niches and possibly even survival in a human host. For example, BtCDC3015869 (also known as TXDOH or 2003015869) represents the only convincing example of Bt human infection in the literature, albeit associated with an extreme scenario of near drowning in a young infant, with subsequent rapid clinical recovery [42], and BtCDC2721121 was isolated from the pleural wound of a 76 year old male from Louisiana in 1997. Since exposures to new niche environments are a well-known evolutionary force for the development of novel functional adaptations [52,53], an enhanced propensity of the outlier strains to persist in novel geographical or ecological niches might have facilitated the gain of subsequent genetic modifications, which may have further enhanced virulence potential. Notably, our data do not preclude the possibility that some of these modifications may have involved the Bp-likeCPS cluster itself. For example, there are >400 non-synonymous SNPs between the Bp-likeCPS and Bp CPS clusters, of which approximately 25% are predicted to cause non-conservative amino acid changes in the resulting proteins. Such subtle alterations could also have contributed to slight differences in capsular polysaccharide structure and ultimately enhanced Bp virulence. Addressing this will require detailed structural analyses of the Bp and Bp-likeCPS products.

## Conclusions

We have identified the existence of a unique set of variant Bt strains carrying molecular features of Bp, and were also able to demonstrate, for the first time, the presence of distinct phylogenetic clades in Bt. Our study suggests that many virulence genes already exist as part of the natural genomic reservoir available for microbial recombination and lateral gene transfer (the species 'pan-genome'). We note that compared to clinical pathogens, comparatively less attention has been paid to the large-scale genomic analysis of non-pathogens. Expanded genomic surveys of both pathogenic and non-pathogenic *Burkholderia *species sampled from diverse regions and isolation sources might allow further relationships among different virulence modifications to be studied, and may prove useful in reconstructing the genetic series of events associated with pathogen evolution.

## Materials and methods

### Ethics declaration

Mouse infection assays were conducted according to national and international guidelines at the Defense Medical and Environmental Research Institute (DSO National Laboratories) in Singapore under Institutional Animal Care and Use Committee (IACUC) protocol number DSO/IACUC/07/42.

### Bacterial strains and genomic DNA extraction

Bacterial cultures were grown in LB media and genomic DNA was extracted using the Qiagen Genomic DNA kit (Qiagen, Hilden, Germany). For morphological characterization, strains were cultured on Ashdown medium at 37°C for 5 days. Appropriate species assignments were confirmed by MLST analysis in this paper and [19] and microbial phenotypes for Thailand strains (colony morphology, resistance to oxidase, colistin and gentamicin, and assimilation of arabinose). Bt and Bp strains were maintained in biosafety level environments BSL2 and BSL3, respectively. BtE555 was also tested for antimicrobial susceptibility using a disk diffusion assay with the exception of co-trimoxazole, which was assessed using an E-test, and shown to be susceptible to amoxicillin-clavulanate, ceftazidime, doxycycline, imipenem and co-trimoxazole.

### *Burkholderia *pan-genome array construction

The BPGA contains 22.3 Mb of genomic sequence derived from 23 Bp strains, four Bt strains, and one *Burkholderia cenocepacia *(Bc) strain (Additional file 1). Bp and Bt pan-genomes were inferred by subjecting the strains to a species-specific analysis pipeline (Additional file 2). First, we aligned one strain genome to the appropriate species reference genome (K96243 for Bp and BtE264 for Bt) using NUCmer [54]. Novel regions of non-synteny were identified and concatenated to the reference genome, forming a working pan-genome. Second, a new strain of the same species was then aligned to this working pan-genome, and additional novel sequences were further incorporated. This process was iteratively repeated for all strains in that species, resulting in a 12.34-Mb Bp and 7.35-Mb Bt pan-genome. Strains were aligned following the order in Additional file 1; however, the specific strain order does not influence the final pan-genome size or composition (Additional file 3). For *Bc*, we performed a reciprocal BLAST (BlastN, cutoff ≥ 1e^-6^) analysis between all Bc J2315 and Bp K96243 genes to identify 3,019 Bc specific genes out of 7,366, which were also added to the BPGA. BPGAs of 392,135 50-mer probes were constructed by maskless photolithography (NimbleGen, Reykjavik, Iceland). Besides *Burkholderia*-related probes (366,064), the BPGA also contains 6,071 control probes of matching GC content, melting temperature, and length, and 20,000 random sequence probes for background estimation and correction. The final composition of the BPGA is shown in Additional file 2.

### Array-based comparative genomic hybridization and data analysis

Genomic DNAs were labeled with either Cy3 or Cy5 fluorescence dyes using a bacterial artificial chromosome (BAC) array labeling kit (Kreatech, BV, Netherlands). Two micrograms of labeled genomic DNA per strain was hybridized to the BPGA at 52°C for 16 h in a MAUI hybridization system (BioMicro Systems, Inc, Salt Lake City Utah, USA). After hybridization and washing, arrays were scanned on an Axon 4000B scanner (Molecular Devices, Sunnyvale, CA, USA). Signal intensity files, generated by NimbleScan version 2.2 (NimbleGen Systems, Madison, WI, USA), were normalized by the LOWESS procedure (R console, Bioconductor package) [55]. Technical validation of the array is presented in Additional files 4 and 5. To detect regions of difference between the Bt isolates and the BtE264 reference genome, log_2 _ratios of Bt isolate signals against BtE264 signals were processed using CGHScan 1.0 [56] using a cutoff of log_2 _ratio ≤ (median - 2 × standard deviation, significance level at 0.05). Recurrent regions of difference were defined as regions exhibiting variability in at least 10% of strains (n ≥ 5). Circular chromosomal diagrams were generated using Circos [57]. The array data were also visualized using SignalMap v 1.9.0.03 (NimbleGen Systems, Inc.). Microarray data and platform details have been deposited in the Gene Expression Omnibus (GEO) database under accession number [GEO:GSE18766].

### BtE555 genome sequencing

BtE555 genomic DNAs were processed for paired-end library construction and sequenced using a Genome Analyser II instrument (Illumina, San Diego, CA, USA). We mapped 100-bp paired-end reads to the reference BtE264 genome initially with ELAND (Illumina) for quality recalibration, and subsequently with Maq-0.7.1 under default parameters [58]. Sequence calls by Maq were filtered to coordinates with ≥20× coverage after PCR duplicate removal. Candidate SNPs were filtered using the following rules: (1) discard SNPs within 5 bp of a potential indel; (2) discard SNPs in reads of mapping quality <60; (3) discard SNPs in any 10-bp window containing three or more SNPs; and (4) discard SNPs with consensus quality calls of <60. SNPs occurring in predicted GIs and tandem repeats were excluded. Functional classification of SNPs was performed using gene co-ordinates from the BtE264 reference genome. *De novo *assembly of the BtE555 paired-end reads was performed using PE-Assembler, an in-house toolkit (PA and WS, manuscript in preparation). *De novo *assembled contigs were mapped to the reference Bt E264 genome using Nucmer [54] and Contig Aligner [59]. Genes on contigs were predicted using FGENESB [60]. The BtE555 sequence data are available in NCBI [GenBank:AECN00000000].

### Phylogenetic analysis (aCGH and MLST)

aCGH phylogenetic trees were based on regions of variability detected by CGHScan, and generated using Ward's hierarchical clustering method and the Hamming distance between strains. Cluster robustness was assessed by perturbing the aCGH data by either removing all STs represented by single strains or randomly deleting 15% of the BtE264 probes over 100 iterations, and computing consistencies between the perturbed and original clusters using Kappa statistics (Additional file 15). MLST was performed as previously described [35] on all 92 Bt isolates (Additional file 17), including 10 previously described Bt isolates [45] and 4 Bt isolates by *in silico *analyses of genome sequences. Network reconstruction of the Bt genotypes was performed using Splitstree 4.10 software [61]. In total, 17 out of 21 STs are shared between the aCGH and MLST analyses. Concordance between aCGH and MLST clusters was determined based on correlating the segregation of specific STs within aCGH clusters, and the degree of ST locus variance within each aCGH cluster (Additional file 18) [40].

### Western blotting and immunofluorescence

Crude extracts from strains were prepared as described previously [62] and resolved using 10% SDS-PAGE. The presence of EPS was examined by immunoblotting to a nitrocellulose membrane and probing with monoclonal antibody 4B11, which is specific to Bp CPS [36], followed by a secondary horseradish peroxidase-conjugated rabbit anti-mouse immunoglobulin (Dako A/S, Glostrup, Denmark). Detection was performed using a chemiluminescence reagent (Roche Diagnostics GmbH, Mannheim, Germany). For immunofluorescence studies, log-phase bacteria were pelleted and resuspended in phosphate-buffered saline (PBS). Primary antibody incubations were performed at 37°C for 30 minutes in 1 ml of hybridoma supernatant containing the 4B11 monoclonal antibody at a dilution of 1:10. After pelleting and multiple washings with 0.05% PBS-Tween20, secondary incubations were performed in 100 μl with Cy5 AffiniPure Fab Fragment Donkey anti-mouse IgG (Jackson Immuno Laboratory, West Grove, PA, USA) at 1:400 dilutions for 30 minutes at 37°C. After pelleting and multiple washings with 0.05% PBS-Tween20, bacteria were then fixed with 1 ml of 2.5% paraformaldehyde for 30 minutes at 37°C prior to staining with DAPI (1:2,000 dilutions from 20 mg/ml solution; Sigma-Aldrich Pte Ltd, Singapore) to visualize nuclei. Samples were visualized on an Axiovert 200 m inverted microscope (Carl Zeiss) fitted with an x-cite 120 Fluorescence Illumination system (EXFO, Mississauga, Ontario, Canada), and a Photometric Coolsnap HQ high sensitivity system (Roper Scientific, Friedland, Germany). Images were analyzed by Metamorph software (Molecular Devices Corporation, Downingtown, PA, USA).

### Targeted disruption of the Bp-likeCPS cluster

The BtE555 CPS cluster was disrupted by an insertional mutagenesis vector targeting the *wcbB *gene. Two fragments (sizes of 794 and 803 bp) upstream and downstream of positions 3,357,307 to 3,357,534 in *wcbB *were PCR amplified from BtE555 genomic DNA, ligated to one another, and cloned into the suicide vector pDM4 [63]. This vector (*wcbBdel*) was introduced into BtE555 by conjugation with a λpir^+ ^donor *Escherichia coli *strain. Recombinants were selected on LB plates supplemented with chloramphenicol (50 μg/ml) and gentamicin (25 μg/ml). Strains exhibiting a recombination of the *wcbBdel *vector into the endogenous *wcbB *gene were confirmed by subsequent PCR analysis and sequencing.

### Complement deposition assay

Log-phase bacteria were pelleted and incubated in 10% normal human serum in a final 1 ml volume at 37°C for 15 minutes. Opsonization was terminated by the addition of EGTA (final concentration of 10 mM). After three rounds of pelleting and washing with 1× PBS, bacterial pellets were fixed in 1 ml 2.5% paraformaldehyde. Blocking was performed in 200 μl of PBS-0.05% Tween 20 overnight at 4°C. Primary antibody incubations were performed at 37°C for 30 minutes in 20 μl of 0.05% PBS-Tween20 with a monoclonal antibody to human complement C3b-alpha (1:20 dilutions from 50 μg/ml; Autogen Bioclear UK Ltd, Holly Ditch Farm, Mile Elm, Calne, Wilts, UK). After washing, secondary antibody incubations and imaging studies were performed as described above (see Immunofluorescence).

### Macrophage infection assay

We seeded 24-well flat-bottom plates with approximately 1 million RAW macrophage cells per well in DMEM supplemented with fetal bovine serum (Invitrogen, Gibco, Grand Island, NY, USA) and these were allowed to adhere overnight. The adhered cells were washed twice with PBS and exposed to log-phase bacteria at a 10:1 multiplicity of infection (MOI). After a 1-h infection period at 37°C, extracellular bacteria were killed by replacing the medium with DMEM supplemented with fetal bovine serum and kanamycin (250 μg/ml). At 2, 4, 6, and 8 hours post-infection, viable intracellular bacteria were harvested by incubating the cells with 0.1% Triton-X100 at 37°C for 30 minutes, and quantified by counting colony forming units (cfu) on plates serially diluted with lysate. All experiments were performed in duplicate on separate days. *P*-values between different strains were calculated using a two-tailed *t*-test.

### Virulence assays in mice and *C. elegans *infection models

Intranasal mouse infection assays were performed as previously described in [45]. For each bacterial strain, 30 BALB/c mice in groups of six were infected intra-nasally with either Bp (5 to 3,600 cfu) or Bt (10^3^-10^7 ^cfu). One group was inoculated with PBS as a negative control. Infected mice were monitored for survival at 4 and 13 days post-infection. LD50 values were then calculated based on day 13 survival values using the method of [64]. *C. elegans *assays were performed as described in [44]. Forty L4-staged wild-type N2 Bristol *C. elegans *were picked and exposed to a 100-μl bacterial lawn. Infected animals were subsequently observed over a 5-day period.

## Abbreviations

ACGH: array-based comparative genomic hybridization; BC: *Burkholderia cenocepacia*; BP: *Burkholderia pseudomallei*; Bp CPS: capsular polysaccharide gene cluster in *Burkholderia pseudomallei*; BP-LIKECPS: capsular polysaccharide gene cluster in variant strains of *Burkholderia thailandensis *(BtE555 and BtCDC3015869), with high nucleotide and protein similarity to Bp CPS; BPGA: *Burkholderia *pan-genome array; BT: *Burkholderia thailandensis*; CFU: colony forming unit; CPS KO: genetic mutants of BtE555 with Bp-likeCPS disrupted; DMEM: Dulbecco's modified Eagle's medium; EPS: exopolysaccharide; GI: genomic island; KO: knockout; MLST: multi-locus sequence typing; NGI: novel genomic islet; PBS: phosphate-buffered saline; SNP: single nucleotide polymorphism; ST: sequence type as defined by MLST.

## Authors' contributions

SP and PT conceived the study. BS participated in creating the BPGA, performed molecular genetic studies, and drafted the manuscript. NC, VW, JT, ST, SP and MG provided strains, and performed immunoassays and MLST studies. WO and EF performed phylogenetic and statistical analysis. TN, RT, PA and WS participated in sequence alignment and SNP identification. XS performed *C. elegans *experiments. HC and KR participated in BtE555 sequencing; CL created and validated the BPGA. YL and GT performed mouse infection assays. EF, GT, SP and PT provided critical analysis, participated in the design, coordination and helped to draft the manuscript. All authors read and approved the final manuscript.

## Supplementary Material

Additional file 1 **A list of the *Burkholderia *strains used to construct the *Burkholderia *pan-genome array (BPGA)**. Click here for file

Additional file 2 **A flowchart that depicts the analysis workflow for the *Burkholderia *pan-genome array (BPGA)**. Click here for file

Additional file 3 **A graph illustrating the robustness of the workflow**. Click here for file

Additional file 4 **A document explaining the experimental validation of the BPGA**. Click here for file

Additional file 5 **A figure that shows the results obtained from the experimental validation of the BPGA**. Click here for file

Additional file 6 **A document validating BtE555 as a Bt species by *ara *gene PCR and 16 s sequencing**. Click here for file

Additional file 7 **A chart that summarizes the average variability in each Bt strain compared against the BtE264 reference**. Click here for file

Additional file 8 **A complete list of all the GIs and nGis and their associated features**. Click here for file

Additional file 9 **Six representative dot matrix plots of the Bp-likeCPS from BtE555 when aligned against Bp CPS from BpK96243**. Click here for file

Additional file 10 **Detailed information of the GC composition of Bt EPS and the Bp-likeCPS in BtE555**. Click here for file

Additional file 11 **Experimental validation of 50 representative SNPs predicted in BtE555**. Click here for file

Additional file 12 **Sequence statistics of the *de novo *assembled contigs from BtE555 deep sequencing paired-end reads**. Click here for file

Additional file 13 **A table that ascribes BtE555 contigs that are mappable to the GI, nGi and EPS regions in BtE264**. Click here for file

Additional file 14 **A list of the BtE555 unique genes and their associated contigs, when compared against reference strain BtE264**. Click here for file

Additional file 15 **Two phylogenetic trees drawn with permuted data, to ensure that the aCGH clusters are robust**. Click here for file

Additional file 16 **List of genes absent in three of the strains in cluster 1 (which contains the variant strains) when compared against BtE264**. Click here for file

Additional file 17 **A list of strains used for both MLST and aCGH analysis**. Click here for file

Additional file 18 **A figure showing the correlation between aCGH similarity and locus variance**. Click here for file

Additional file 19 **A rooted phylogenetic tree (MLST) confirming that the variant strains are distinct from the rest of the Bt strains**. Click here for file

Additional file 20 **A graph that charts the growth rate of the reference strain BtE264, BtE555 and the mutant CPS KO in rich media**. Click here for file
