# Supplementary material for: Genomic acquisition of a capsular polysaccharide virulence cluster by non-pathogenic Burkholderia isolates
Source: Genome Biol. 2010 Aug 27;11(8):R89. doi: 10.1186/gb-2010-11-8-r89 (PMC2945791; doi:10.1186/gb-2010-11-8-r89)
Supplement: Additional file 1 — A list of the Burkholderia strains used to construct the Burkholderia pan-genome array (BPGA). [file gb-2010-11-8-r89-S1.DOC]

**Additional data file 1. *Burkholderia* strains used to construct the Burkholderia pan-genome array (BPGA).**

| Strain | Number of contigs / chromosome | Total sequence length | Gen Bank Accession Numbers | Number of novel genomic fragments added onto BPGA | Total genomic sequence on BPGA |
| --- | --- | --- | --- | --- | --- |
| ***B. pseudomallei K96243*** | **2** | **7247547** | **BX571965, BX571965** | **Reference Genome** | **7247547** |
| *B. pseudomallei 9* | 1762 | 6805947 | ABBL01000001 - ABBL01001762 | 90 | 261065 |
| *B. pseudomallei 14* | 1888 | 6707583 | ABBJ01000001 - ABBJ01001888 | 48 | 130394 |
| *B. pseudomallei 22* | 4 | 6699567 |  | 64 | 115165 |
| *B. pseudomallei 91* | 1690 | 6867787 | ABBK01000001 - ABBK01001690 | 13 | 62304 |
| *B. pseudomallei 112* | 1274 | 6919035 | ABBP01000001 - ABBP01001274 | 45 | 129454 |
| *B. pseudomallei 305* | 36 | 7453649 | AAYX01000001 - AAYX01000036 | 104 | 427872 |
| *B. pseudomallei 381* | 1230 | 6956166 | ABBM01000001 - ABBM01001230 | 1390 | 1971636 |
| *B. pseudomallei 406e* | 271 | 7322716 | AAMM02000001- AAMM02000271 | 98 | 155849 |
| *B. pseudomallei 668* | 2 | 7040403 | CP000570 - CP000571 | 81 | 167985 |
| *B. pseudomallei 1106a* | 2 | 7089249 | CP000572 - CP000573 | 27 | 80019 |
| *B. pseudomallei 1106b* | 113 | 7213434 | AAMB02000001 - AAMB02000113 | 95 | 114634 |
| *B. pseudomallei 1655* | 196 | 7029452 | NZ_AAHR01000001 - NZ_AAHR01000196 | 54 | 81546 |
| *B. pseudomallei 1710a* | 209 | 7319487 | NZ_AAHS01000001 - NZ_AAHS01000209 | 75 | 146024 |
| *B. pseudomallei 1710b* | 2 | 7308054 | NC_007434 - NC_007435 | 14 | 8648 |
| *B. pseudomallei 7210* | 1424 | 6891693 | ABBN01000001 - ABBN01001424 | 16 | 138292 |
| *B. pseudomallei 7894* | 1568 | 6978293 | ABBO01000001 - ABBO01001568 | 54 | 154445 |
| *B. pseudomallei BCC215* | 1030 | 7000410 | ABBR01000001 - ABBR01001030 | 36 | 94082 |
| *B. pseudomallei Dm98* | 2371 | 6688656 | ABBI01000001 - ABBI01002371 | 43 | 84129 |
| *B. pseudomallei NCTC13177* | 1077 | 7123770 | ABBQ01000001 - ABBQ01001077 | 48 | 119775 |
| *B. pseudomallei Pasteur* | 228 | 7346573 | NZ_AAHV01000001 - NZ_AAHV01000228 | 60 | 173810 |
| *B. pseudomallei S13* | 179 | 7388320 | NZ_AAHW01000001 - NZ_AAHW01000179 | 63 | 237201 |
| *B. pseudomallei SAVP* | 93 | 5283468 | NZ_AAHQ01000001 - NZ_AAHQ01000093 | 198 | 234788 |
| ***B. thailandensis E264*** | **2** | **6723972** | **NC_007651 , NC_007650** | **Reference Genome** | **6723972** |
| *B. thailandensis 4* | 803 | 6555995 | ABBH01000001 - ABBH01000803 | 64 | 242189 |
| *B. thailandensis 700388* | 2 | 6304274 | CM000438 - CM000439 | 5 | 1601 |
| *B. thailandensis CDC3015869* | 810 | 6515911 | ABBD01000001 - ABBD01000810 | 177 | 385292 |
| ***B. cenocepacia J2315*** | **3** | **7963121** | **ftp: ftp.sanger.ac.uk/pub/bc** | **3019 (individual genes)** | **2642624** |

**Additional data file 1. *Burkholderia* strains used to construct the Burkholderia pan-genome array (BPGA).**

The three reference genomes (BpK96243, BtE264, and BcJ2315 are highlighted in bold type. Most genomes are partially completed, as reflected by the presence of multiple contigs (column 2). BtCDC3015869 is also known as TXDOH or 2003015869. The number of novel genomic regions and amount of novel genome sequence from each strain after iterative alignment to the working pan genome is listed here (column 5 and 6), adding up to a final pan genome size of 22.3 Mb from 28 *Burkholderia* strains.
