# Supplementary material for: Genomic acquisition of a capsular polysaccharide virulence cluster by non-pathogenic Burkholderia isolates
Source: Genome Biol. 2010 Aug 27;11(8):R89. doi: 10.1186/gb-2010-11-8-r89 (PMC2945791; doi:10.1186/gb-2010-11-8-r89)
Supplement: Additional file 2 — A flowchart that depicts the analysis workflow for the Burkholderia pan-genome array (BPGA). [file gb-2010-11-8-r89-S2.DOC]

**Additional data file 2. Analysis workflow for the *Burkholderia* pan genome array (BPGA).**

**
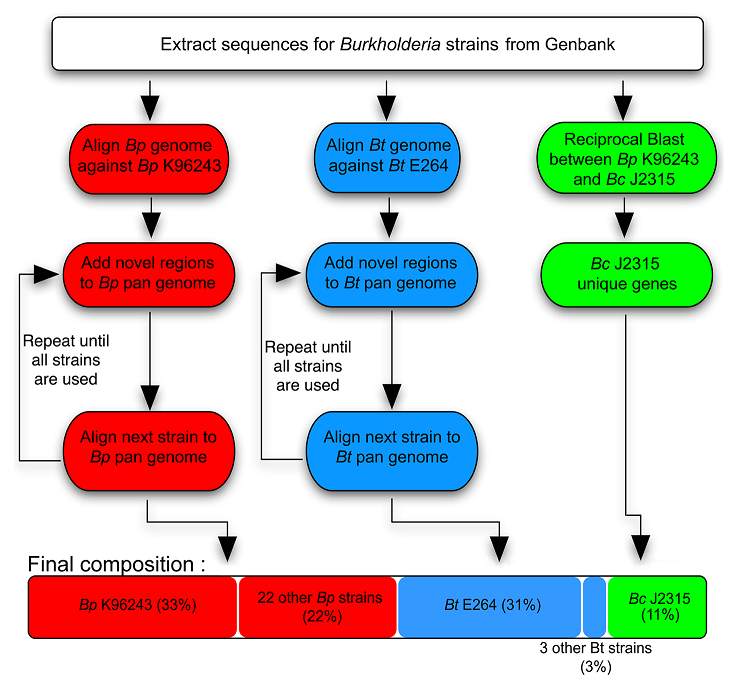
**

**Additional data file 2. Analysis workflow for the *Burkholderia* pan genome array (BPGA).** Genome sequences were processed using three independent species-specific pipelines (Bp, Bt and Bc). For the Bp genomes, each Bp strain genome was aligned, first against the BpK96243 reference genome, and subsequently against an expanding Bp pan genome, to identify novel sequences. A similar pipeline was used to define the Bt pan genome. For Bc, we identified genes in BcJ2315 not found in BpK96243 by a reciprocal Blast protocol (3019 Bc-specific genes). These Bc specific genes were also added to the pan genome array. The final composition of BPGA used for array construction (total length = 22.3 Mb) comprises of 12.34 Mb (55%) of non-redundant sequence from 23 Bp strains (BpK96243 and 22 additional Bp genomes, red bar). The Bt pan-genome comprises 7.35 Mb (34%) of non-redundant sequence from 4 Bt strains (Bt E264 and 3 additional Bt genomes, blue bar). The array also contains 2.64 Mb (11%) of additional Bc-specific sequence found in Bc J2315 (green) but not Bp K96243. See Main Text for details on array construction.
