# Supplementary material for: Genomic acquisition of a capsular polysaccharide virulence cluster by non-pathogenic Burkholderia isolates
Source: Genome Biol. 2010 Aug 27;11(8):R89. doi: 10.1186/gb-2010-11-8-r89 (PMC2945791; doi:10.1186/gb-2010-11-8-r89)
Supplement: Additional file 3 — A graph illustrating the robustness of the workflow. [file gb-2010-11-8-r89-S3.DOC]

**Additional data file 3. Order of individual strains does not influence final pan genome size or composition.**


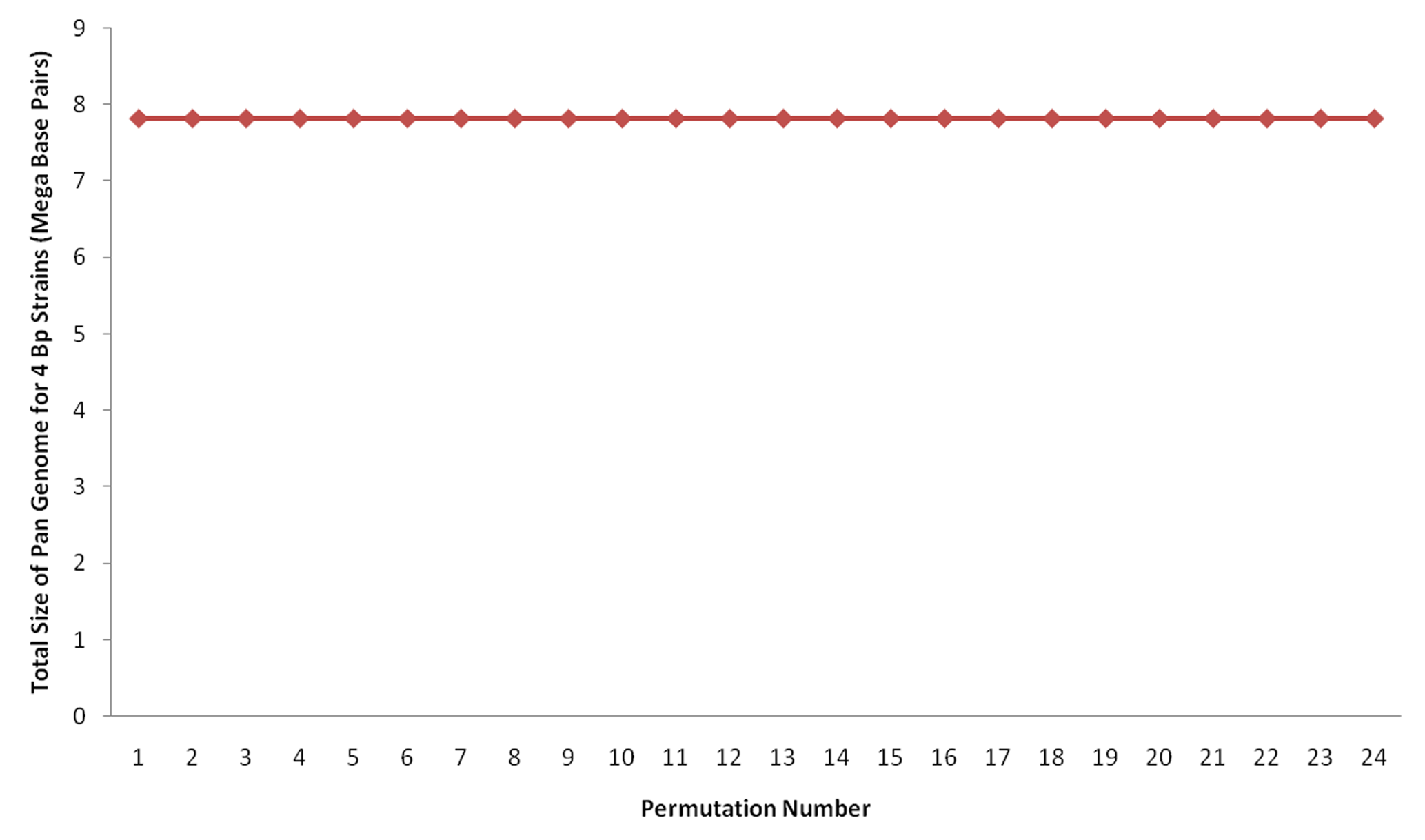


**Additional data file 3. Order of individual strains does not influence final pan genome size or composition.** Four Bp strains were randomly selected, and subjected to the analysis pipeline of Additional data file 2. The specific order of the Bp strains was then permuted, and the same strains (now in permuted order) were subjected to the same pipeline. This was repeated for all 24 possible permuted orders (x-axis). A comparison of the final size of the pan-genome outputs confirms that the final pan-genome composition is independent of strain order.
