# Supplementary material for: Genomic acquisition of a capsular polysaccharide virulence cluster by non-pathogenic Burkholderia isolates
Source: Genome Biol. 2010 Aug 27;11(8):R89. doi: 10.1186/gb-2010-11-8-r89 (PMC2945791; doi:10.1186/gb-2010-11-8-r89)
Supplement: Additional file 4 — A document explaining the experimental validation of the BPGA. [file gb-2010-11-8-r89-S4.DOC]

**Additional data file 4. Experimental Validation of the BPGA**

To experimentally validate this platform, we hybridized to the BPGAs a set of genomic DNAs from sequenced strains, and considered the array results against independent predictions from actual nucleotide-level sequence comparisons. We compared strains belonging to the same species (Bp K96243 vs Bp 22) or from different species (Bp K96243 vs Bt E264) (see Additional file 5). After lowess normalization, signal profiles for the Cy3 and Cy5 channels were partitioned into two clusters representing either background signals (noise) or true probe signal, using a classification method involving normal mixture modeling and model-based clustering (MCLUST v3). Probe signals two standard deviations (SD) above the mean of the background signal peak were considered as true signals (TS). Probes exhibiting TS signals in both channels were designated as “common” to both strains, while strains exhibiting TS signals in one channel and not the other were designated as “strain-specific”. We then compared these subsets of probes to known sequence data. In the Bp K96243 vs Bp 22 comparison (same species), >95% of BPGA probes designated as “common” to both strains were indeed conserved in both strains at the sequence level. In contrast, 98.9% of BPGA probes designated as “K96243-specific” corresponded to sequences found in Bp K96243 and not Bp 22, and 95.2% of BGPA probes designated as “Bp 22-specific” corresponding to sequences in Bp 22 and not Bp K96243. Similar results were obtained in the Bp K96243 vs Bt E264 comparison (different species), with 99.8% and 98.5% of “K96243-specific” and “E264-specific” probes corresponding to sequences found exclusively in one genome and not the other. Discrepancies between the BPGA hybridization results and genome sequence comparisons could be attributable to the use of highly stringent match requirements in the BLAST sequence analysis (100% match for an initial seed of 11 nucleotides). Taken collectively, these results verify the ability of the BPGA to rapidly identify genomic regions of species and strain-specificity in a single hybridization experiment.
