# Supplementary material for: Genomic acquisition of a capsular polysaccharide virulence cluster by non-pathogenic Burkholderia isolates
Source: Genome Biol. 2010 Aug 27;11(8):R89. doi: 10.1186/gb-2010-11-8-r89 (PMC2945791; doi:10.1186/gb-2010-11-8-r89)
Supplement: Additional file 5 — A figure that shows the results obtained from the experimental validation of the BPGA. [file gb-2010-11-8-r89-S5.DOC]

**Additional data file 5. Experimental validation of the *Burkholderia* pan genome array.**

**
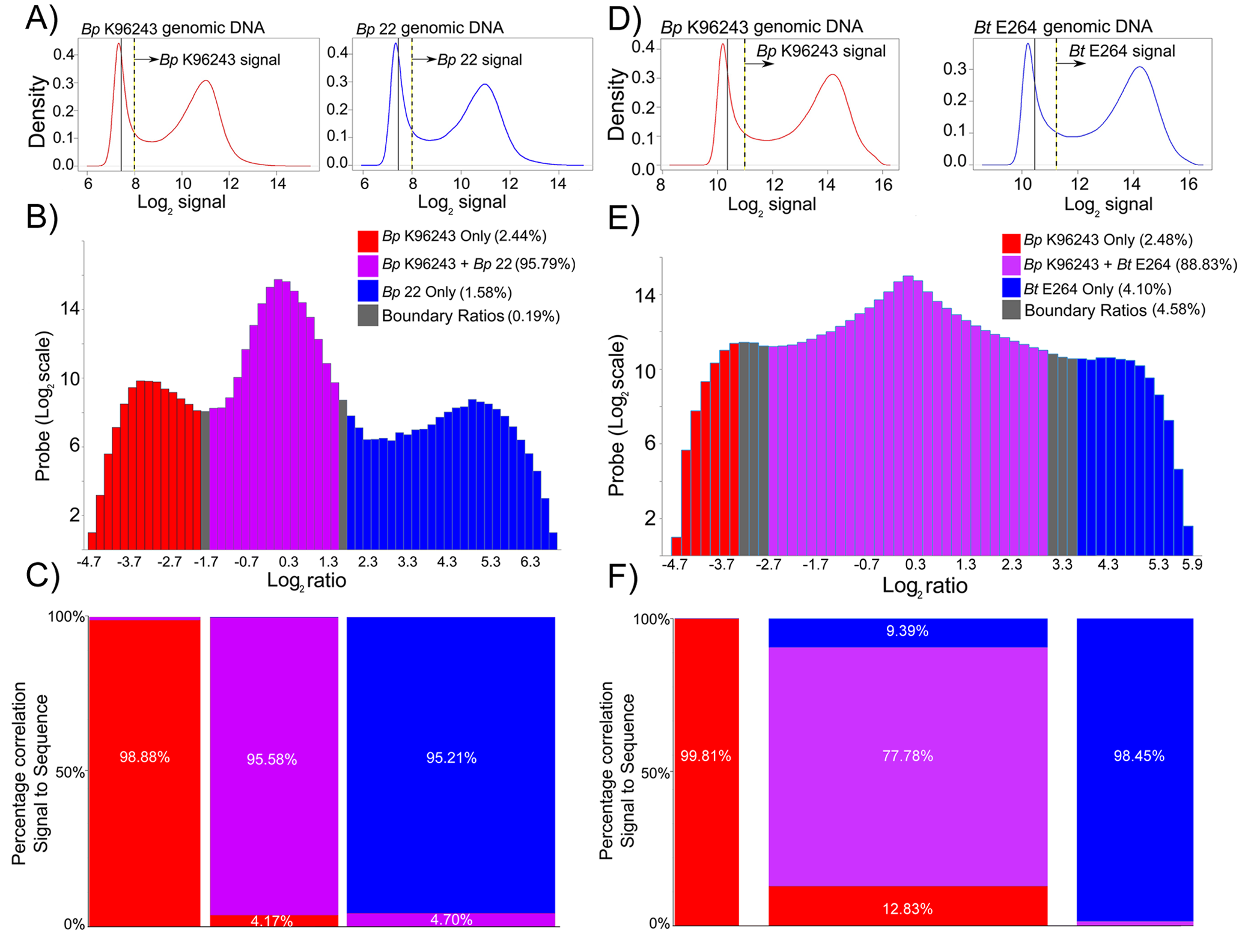
**

**Additional data file 5.** **Experimental validation of the *Burkholderia* pan genome array.** The pan genome array was validated by hybridizing genomic DNAs from strains of known genomic sequence onto the array.

(A-C) Results from hybridization of Bp K96243 against Bp 22 genomic DNA (intra-species). Both BpK96243 and Bp22 genomic DNA were hybridized onto the pan genome microarray in a dual channel experiment.

A) Hybridization signals associated with BpK96243 (left) and Bp22 (right) genomic DNA. Raw flouresence data was processed by MCLUST (see additional data file 4). Each hybridization profile occurs as a two-peak signal distribution, corresponding to background flouresence intensities (left peak) and true signal-associated flouresence intensities (right peak). A cut-off corresponding to 2 standard deviations (log_2_ signal of approximately >8) above the background peak mean was used to define probes exhibiting true signals. Signals from these probes were then extracted for subjected to further analysis (B and C).

B) Probes exhibiting true signals were subdivided into those showing (red) signals only in the Bp K96243 hybridization, (purple) signals in both the BpK96243 and Bp22 hybridization, and (blue) signals only in the Bp22 hybridization. Y-axis : Probe abundance (log_2_ scale). X-axis : Hybridization ratios of probes. “Common” probes correspond to probes exhibiting true signals (>2 SD) in both strains (purple), while probes exhibiting true signals in one strain and not the other were considered as “strain-specific” (red and blue). Probes at the boundaries of the common and strain specific regions were designated as ambiguous and remove from further analysis (grey).

C) Comparison between experimental hybridization results and predictions based on computational sequence analysis. (left column) 98.88% of BpK96243 specific probes (determined by hybridization) correspond to BpK96243 specific sequence predicted by computational analysis. (middle column) 95.58% of BpK96243 and Bp22 common probes (determined by hybridization) correspond to sequences found both in Bp K96243 and Bp 22. (right column) 95.21% of Bp 22 specific probes (determined by hybridization) correspond to Bp 22 specific sequence predicted by computational analysis. Discrepancies (4.17% in middle column, 4.7% in right column) largely correspond either to regions of short sequence similarity between the strains or regions of sequence ambiguity (NNN) in the Bp 22 genome.

(D-F) Results from hybridization of BpK96243 against BtE264 genomic DNA (inter-species). Both BpK96243 and BtE264 genomic DNA were hybridized onto the pan genome microarray in a dual channel experiment.

D) Hybridization signals associated with BpK96243 (left) and BtE264 (right) genomic DNA. See panel A) for details.

E) Probe distributions of common and strain-specific probes. Red probes correspond to probes showing true signals only in the BpK96243 hybridization, purple probes to those common to both the BpK96243 and BtE264 hybridization, and blue probes those probes exhibiting true signals only in the BtE264 hybridization. See panel B) for details.

F) Comparison between experimental hybridization results and predictions based on computational sequence analysis. (left column) 99.81% of BpK96243 specific probes (determined by hybridization) correspond to BpK96243 specific sequence predicted by computational analysis. (middle column) 77.78% of BpK96243 and BtE264 common probes (determined by hybridization) correspond to sequences found both in BpK96243 and BtE264. (right column) 98.45% of BtE264 specific probes (determined by hybridization) correspond to Bt E264 specific sequence predicted by computational analysis. For the middle column, discrepancies between probes called as common by hybridization vs species-specific by sequence (9.39% and 12.83%) respectively arise due to the stringent BLAST parameters used (11 word window, perfect match) to assign common probes, which would result in highly related but not identical sequences called as species-specific rather than common.
