# Supplementary material for: Genomic acquisition of a capsular polysaccharide virulence cluster by non-pathogenic Burkholderia isolates
Source: Genome Biol. 2010 Aug 27;11(8):R89. doi: 10.1186/gb-2010-11-8-r89 (PMC2945791; doi:10.1186/gb-2010-11-8-r89)
Supplement: Additional file 6 — A document validating BtE555 as a Bt species by ara gene PCR and 16 s sequencing. [file gb-2010-11-8-r89-S6.DOC]

**Additional data file 6. Confirmation of BtE555 as *B. thailandensis.***


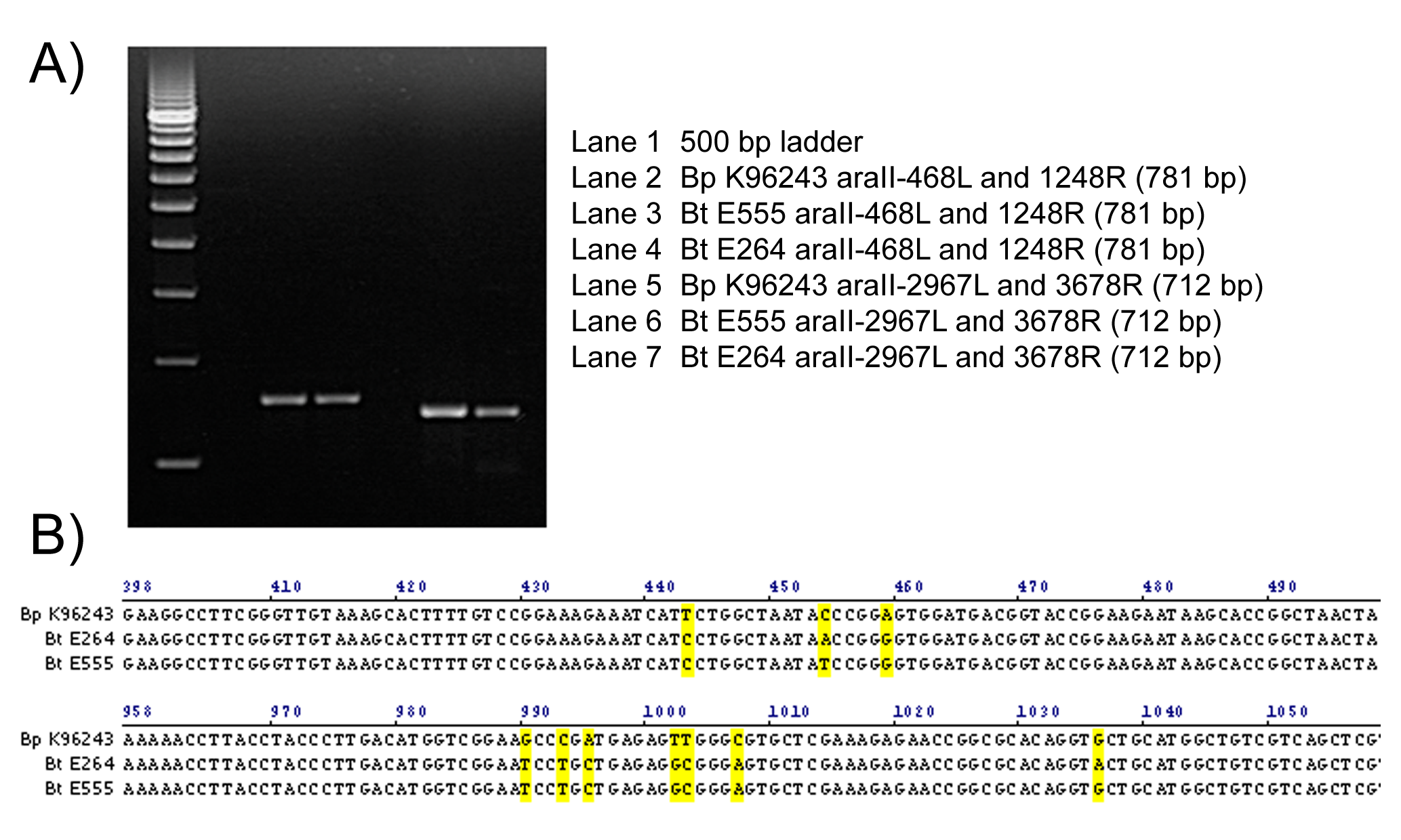


**Additional data file 6. Confirmation of BtE555 as *B. thailandensis.***

A) PCR analysis was performed to confirm the presence of arabinose cluster genes (Chr 2) in BtE555. Two representative genes from the arabinose cluster (BTH_I1626 and BTH_I1628) were tested. The genes are present in BtE555 and BtE264 (lanes 3, 4, 6, 7), but absent in BpK96243 (lanes 2 and 5).

B) 16S rRNA sequences of BpK96243, BtE264, and BtE555. BtE555 was confirmed as a Bt strain by querying the Greengenes BLAST database (top hits : BtE264, Bt str 2003015869, and Bt str 82172) confirms that BtE555 is indeed a Bt strain, similar to E264. Differences between the Bp and Bt strains are highlighted in yellow.
