# Supplementary material for: Genomic acquisition of a capsular polysaccharide virulence cluster by non-pathogenic Burkholderia isolates
Source: Genome Biol. 2010 Aug 27;11(8):R89. doi: 10.1186/gb-2010-11-8-r89 (PMC2945791; doi:10.1186/gb-2010-11-8-r89)
Supplement: Additional file 7 — A chart that summarizes the average variability in each Bt strain compared against the BtE264 reference. [file gb-2010-11-8-r89-S7.DOC]

**Additional data file 7.** **Average variability in each strain**.


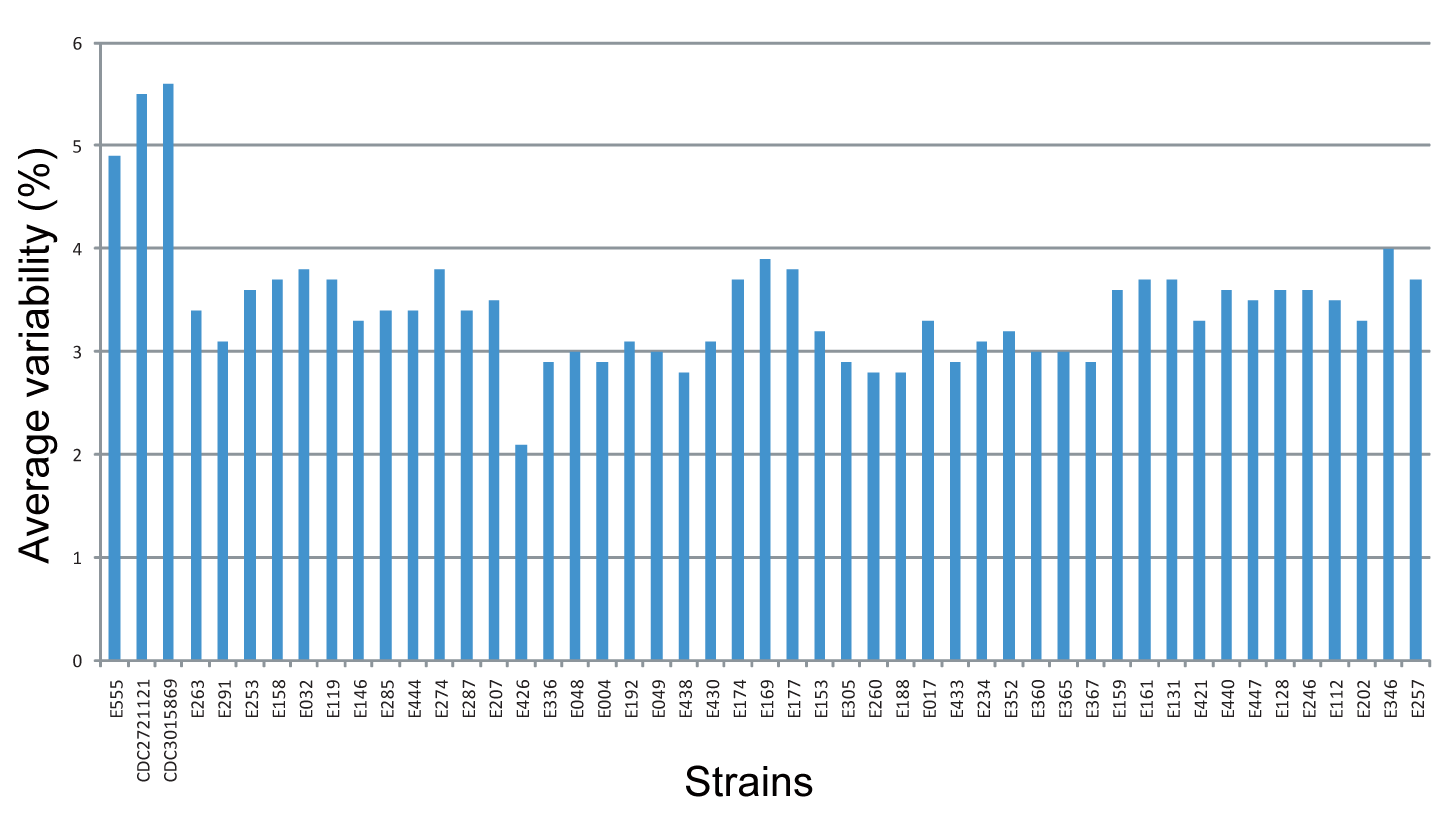


**Additional data file 7.** **Average variability in each strain**. The genomic variability of each strain was determined by CGHScan using a cutoff at median – 2 standard deviation and significance level 0.05, and compared against the total length of both chromosomes. Variability of strains BtE555, BtCDC2721121 and BtCDC3015869 are relatively higher than the other Bt isolates. The average variability in each strain when compared in a pair-wise manner against Bt E264, is approximately 3.4%. Total variability from all strains, when all variable regions are combined account for up to 8% of the BtE264 genome. Note that this index of variability is a lower limit as genomic regions found in isolates but not in BtE264 are not considered.
