# Supplementary material for: Genomic acquisition of a capsular polysaccharide virulence cluster by non-pathogenic Burkholderia isolates
Source: Genome Biol. 2010 Aug 27;11(8):R89. doi: 10.1186/gb-2010-11-8-r89 (PMC2945791; doi:10.1186/gb-2010-11-8-r89)
Supplement: Additional file 8 — A complete list of all the GIs and nGis and their associated features. [file gb-2010-11-8-r89-S8.DOC]

**Additional data file 8. Complete list of all GI and nGis.**

| **ID** | **START** | **STOP** | **LENGTH** | **GC%** | **Integrase** | **prophage, bacteriophage** | **hypothetical** | **transposase, transposon** | **CDS** | **genes within the GI/ nGi** | **genes that are partially**  **in GI/ nGi** |
| --- | --- | --- | --- | --- | --- | --- | --- | --- | --- | --- | --- |
| **CHR I** |  |  |  |  |  |  |  |  |  |  |  |
| **nGi-01** | 79870 | 83100 | 3231 | 56.6 |  |  |  |  | BTH_I0075 | recombinase | stage 0 sporulation protein J |
| **GI-01**  *97241- 148533* | 96279 | 148882 | 52604 | 59.5 | 1 | 1,1 | 21 |  | BTH_I0090-BTH_I0119 | PBSX family phage portal protein, DEAD/DEAH box helicase, XRE family transcriptional regulator, protein kinase domain-containing protein, gp31, gp30 |  |
| **nGi-02** | 559124 | 559510 | 387 | 59.9 |  |  |  |  |  |  |  |
| **nGi-03** | 560626 | 562894 | 2269 | 62.0 |  |  |  |  |  |  | transposase, carboxymuconolactone decarboxylase family protein |
| **nGi-04** | 734736 | 736134 | 1399 | 56.0 | partial |  |  |  |  |  | phage integrase family protein, |
| **nGi-05** | 843311 | 844050 | 740 | 62.2 |  |  |  |  |  |  | ISBma1, transposase |
| **nGi-06** | 920490 | 922039 | 1550 | 58.4 | partial |  | 1 |  | BTH_I0808-BTH_I0809 | transcriptional regulator | integrase protein, TrapT dctQ-M fusion permease, dicarboxylate transport |
| **GI-02**  *1032258- 1050686* | 1037626 | 1050728 | 13103 | 62.6 | 1 |  | 6 | 4 | BTH_I0913-BTH_I0928 | phage related protein, ISBma1, gp11, gp12, lysozyme |  |
| **GI-03**  *1625457- 1652568* | 1627883 | 1653194 | 25312 | 57.7 |  |  | 5 | 2 | BTH_I1442-BTH_I1456 | superfamily I DNA/RNA helicase, HAD superfamily hydrolase, TnpB protein, TnpC protein |  |
| **nGi-07** | 1712344 | 1713715 | 1372 | 58.5 |  |  |  |  |  |  |  |
| **nGi-08** | 1735469 | 1740230 | 4762 | 59.6 |  |  | 1 | 4 | BTH_I1533-BTH_I1538 | helicase, ISBma1 |  |
| **nGi-09** | 1961671 | 1964812 | 3142 | 63.5 |  |  |  | 2 | BTH_I1754-BTH_I1755 | ISBma3 |  |
| **nGi-10** | 2056529 | 2057580 | 1052 | 57.5 |  |  | 1 |  | BTH_I1830 |  |  |
| **GI-04**  *2162503- 2177956* | 2162263 | 2178172 | 15910 | 61.8 | 1 | 1 | 1 | 2 | BTH_I1914-BTH_I1932 | integrative genetic element Gsu32, pyocin R2_PP, tail formation, gp25a, DNA adenine methylase, PAAR motif-containing protein, gp33 | DNA mismatch repair protein MutS |
| **nGi-11** | 2945746 | 2948403 | 2658 | 61.2 |  |  |  | 4 | BTH_I2583-BTH_I2586 | ISBma1 |  |
| **nGi-12** | 3067905 | 3070342 | 2438 | 65.4 |  |  |  |  | BTH_I2687 | manganese transport protein MntH |  |
| **nGi-13** | 3072941 | 3081419 | 8479 | 54.7 |  |  | 2 |  | BTH_I2691-BTH_I2692 |  | Rhs element Vgr protein |
| **GI-05**  *3108624- 3159967* | 3112466 | 3158693 | 46228 | 59.4 | 1 |  | 12 | 2 | BTH_I2717-BTH_I2747 | acetyltransferase, pathogenesis-related protein, outer membrane hemolysin activator protein, filamentous haemagglutinin, plasmid related protein, DNA-binding protein, helix-turn-helix domain-containing protein, TnpC protein, TnpB protein, type I restriction-modification system endonuclease, type I restriction-modification system specificity determinant, type I restriction system adenine methylase, recombinase, stage 0 sporulation protein J | stage 0 sporulation protein J |
| **nGi-14** | 3160005 | 3160851 | 847 | 55.7 |  |  |  |  |  |  |  |
| **nGi-15** | 3253426 | 3254813 | 1388 | 63.3 |  |  |  |  |  |  | ISBma3, transposase |
| **GI-07**  *3416258- 3417080* | 3416597 | 3417443 | 847 | 59.0 |  |  | 1 |  | BTH_I2971 |  |  |
| **GI-08**  *3567805- 3588998* | 3567823 | 3589501 | 21679 | 56.4 | 1  +  partial |  | 4 | 2 | BTH_I3131-BTH_I3143 | DNA-binding protein, resolvase TnpR, helicase domain-containing protein, TnpC protein, TnpB protein | integrase |
| **nGi-16** | 3640233 | 3641589 | 1357 | 63.1 |  |  |  | 1 | BTH_I3194 | ISBma3 |  |
| **GI-09**  *3668227- 3683166* | 3668018 | 3684965 | 16948 | 59.5 | 1 |  | 3 | 1,1 | BTH_I3221-BTH_I3232 | transcriptional regulator, acetyltransferase, ABC transporter, DNA-binding protein BprA, Rhs element Vgr protein, PAAR motif-containing protein | tRNA modification GTPase TrmE |
| **nGi-17** | 3695865 | 3697732 | 1868 | 57.1 |  |  | 2 |  | BTH_I3242-BTH_I3243 |  | DNA gyrase subunit B |
| **GI-10**  *3724755- 3736113* | 3723272 | 3734532 | 11261 | 60.0 | 2 |  | 9 | 2 | BTH_I3264-BTH_I3278 | phage-related secreted protein | helix-turn-helix domain-containing protein, transposase mutator family protein |
| **CHR II** |  |  |  |  |  |  |  |  |  |  |  |
| **nGi-18** | 95866 | 97044 | 1179 | 60.6 |  |  |  |  |  |  | transposase |
| **nGi-19** | 100664 | 101111 | 448 | 58.3 |  |  |  |  |  |  | hypothetical protein BTH_II0090 |
| **nGi-20** | 102523 | 108250 | 5728 | 59.3 | 1 |  | 2 | 2 | BTH_II0091-BTH_II0096 | PAAR motif-containing protein | hypothetical protein BTH_II0090, hypothetical protein BTH_II0097 |
| **GI-11**  *426275- 440945* | 439681 | 441077 | 1397 | 61.8 |  |  |  | 2 | BTH_II0366-BTH_II0367 |  | hypothetical protein BTH_II0368 |
| **nGi-21** | 728435 | 731878 | 3444 | 59.7 |  |  |  | 2 | BTH_II0623-BTH_II0625 | Bbp50 | acyltransferase |
| **nGi-22** | 1100532 | 1101291 | 760 | 58.4 |  |  |  |  |  |  | hypothetical protein BTH_II0937 |
| **GI-12**  *1197987- 1242708* | 1197886 | 1242350 | 44465 | 61.6 | 1 | 0,2 | 15 | 1 | BTH_II1011-BTH_II1069 | gp38, gp44, gp41, gp42, phage protein, gp40-related protein, gp56-related protein, gp49, gp50, gp51, gp52, gp63, gp64, gp65, Mte8-like protein, gp56, gp57, gp58, gp69, gp60, gp72, gp62, gp74, gp64, gp65, 59R, phage terminase, large subunit, head portal protein, ClpP protease, HK97 family phage major capsid protein, phage head-tail adaptor, HK97 family phage protein, gp10, gp11, Phage tail assembly chaperone, Phage minor tail protein, phage minor tail protein L, host specificity protein J, holin, gp23, DNA adenine methylase | major facilitator family transporter, MFS transporter |
| **GI-13**  *1579401- 1614907* | 1578748 | 1614963 | 36216 | 65.1 |  | 5,3 | 14 |  | BTH_II1325-BTH_II1368 | phage-related tail transmembrane protein, phage tail protein P2, gpE+E, phage major tail tube protein, Phage tail sheath protein, phage-related tail fiber protein, phage-related tail protein, baseplate J-like protein, site-specific DNA-methyltransferase, phage virion morphogenesis protein, protein lysB, Phage tail protein X, Phage small terminase subunit, P2 family phage major capsid protein, Phage capsid scaffolding protein (GPO), PBSX family phage portal protein, gp31, gp30, ParA family protein, DNA methyltransferase, gp51 |  |
| **nGi-23** | 1815251 | 1816637 | 1387 | 63.3 |  |  |  |  |  |  | ISBma3, transposase |
| **nGi-24** | 1942600 | 1943870 | 1271 | 61.1 |  |  |  |  |  |  | ISBma1, transposase |
| **GI-15**  *2442484- 2461841* | 2443159 | 2461985 | 18827 | 58.2 | 1 | 1 | 6 | 0,1 | BTH_II1997-BTH_II2013 | gp30, gp31, adenylosuccinate synthase, splicing coactivator subunit-like protein, DNA primase, DNA-binding protein, H-NS histone family protein, DNA-binding protein |  |
| **nGi-25** | 2735798 | 2736055 | 258 | 62.4 |  |  |  |  |  |  | alcohol dehydrogenase BadC |
| **nGi-26** | 2739022 | 2739410 | 389 | 62.5 |  |  |  |  |  |  | drug resistance transporter, EmrB/QacA family protein |

**Additional data file 8. Complete list of all GI and nGis.**

Listed in this Table are 13 previously annotated GIs, and an additional 26 nGis, shown to be recurrently variable in the Bt strain panel. The smallest nGi is 258 bp, and the largest nGi is 8.5 kb (median of 1.4 kb). Boundaries of previously known genomic islands, inferred from computational analysis, are stated under their respective IDs in green type.
