# Supplementary material for: Genomic acquisition of a capsular polysaccharide virulence cluster by non-pathogenic Burkholderia isolates
Source: Genome Biol. 2010 Aug 27;11(8):R89. doi: 10.1186/gb-2010-11-8-r89 (PMC2945791; doi:10.1186/gb-2010-11-8-r89)
Supplement: Additional file 9 — Six representative dot matrix plots of the Bp-likeCPS from BtE555 when aligned against Bp CPS from BpK96243. [file gb-2010-11-8-r89-S9.DOC]

**Additional data file 9. Nucleotide similarities of CPS PCR products from BtE555 compared to BpK96243.**


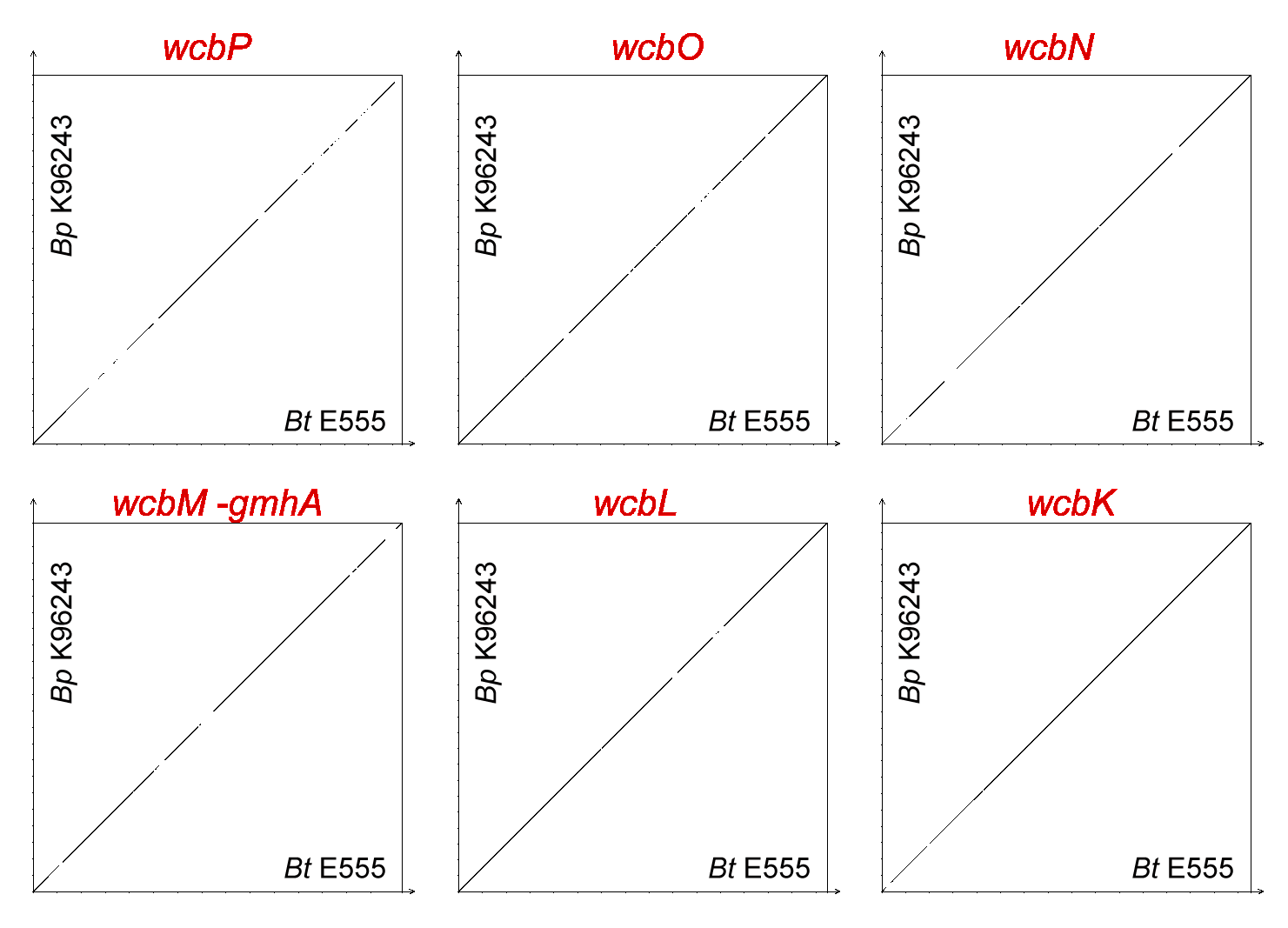


**Additional data file 9. Nucleotide similarities of CPS PCR products from BtE555 compared to BpK96243.** DNA sequences of PCR products amplified from BtE555 genomic DNA were aligned against the homologous BpK96243 Bp CPS genes. Regions of similarity are illustrated with a Dot Matrix plot (Vector NTI suite 10) using a cutoff of ≥90% sequence identity (black spots), and a scanning window of 20 nt.
