# Supplementary material for: Genomic acquisition of a capsular polysaccharide virulence cluster by non-pathogenic Burkholderia isolates
Source: Genome Biol. 2010 Aug 27;11(8):R89. doi: 10.1186/gb-2010-11-8-r89 (PMC2945791; doi:10.1186/gb-2010-11-8-r89)
Supplement: Additional file 10 — Detailed information of the GC composition of Bt EPS and the Bp-likeCPS in BtE555. [file gb-2010-11-8-r89-S10.DOC]

**Additional data file 10. GC Composition of Bt EPS and Bp-likeCPS.**

Figure from Yu et al., 2006 [1]

The Bt variant strain BtE555 has acquired the Bp-likeCPS cluster by replacing the Bt EPS cluster (genes BTH_I1328 to BTH_I1337; annotations derived from the reference BtE264 strain; see above schematic from Yu et al., 2006). The average G+C content of the genes in the Bt EPS cluster is 65.5%, similar to the G+C content of chromosome I (67.3%) (see table below, part a). In contrast, the average G+C content of the Bp-likeCPS cluster genes of Bt E555 is 59.2% (see table below, part b). This compositional skew is also similar to the skew seen between the Bp CPS cluster and the Bp genome [2].

(a) Bt EPS cluster (BTH_I1328 to BTH_I1337)

| **S.No** | **BT-E264** | **Length (bp)** | **GC content (%)** |
| --- | --- | --- | --- |
| 1 | methyltransferase FkbM family protein | 2358 | 62.68 |
| 2 | glycosyltransferase, putative | 1239 | 67.96 |
| 3 | glycosyltransferase WbpX, putative | 1563 | 64.04 |
| 4 | GDP-mannose 4,6-dehydratase | 1038 | 61.85 |
| 5 | GDP-6-deoxy-D-lyxo-4-hexulose reductase, putative | 894 | 65.77 |
| 6 | glycosyl transferase, group 1 family protein, putative | 1032 | 70.54 |
| 7 | glycosyltransferase, putative | 1296 | 71.14 |
| 8 | BexA | 654 | 63.76 |
| 9 | ctrC protein | 795 | 63.27 |
| 10 | WcbD | 1167 | 64.27 |
|  | **Average** | 1167 | 65.5 |

(b) Bp-likeCPS cluster genes in Bt E555

| **S.No** | **BT-E555** | **Length (bp)** | **GC content (%)** |
| --- | --- | --- | --- |
| 1 | wcbT | 1316 | 68.92 |
| 2 | wcbS | 914 | 70.79 |
| 3 | wcbR | 7635 | 69.61 |
| 4 | wcbQ | 1533 | 59.75 |
| 5 | wcbP | 789 | 66.03 |
| 6 | wcbO | 1204 | 58.72 |
| 7 | wcbN | 568 | 58.63 |
| 8 | wcbM | 693 | 58.44 |
| 9 | gmhAshorten | 579 | 59.76 |
| 10 | wcbL | 1041 | 57.35 |
| 11 | wcbK | 1014 | 58.09 |
| 12 | wcbJ | 843 | 57.18 |
| 13 | wcbI | 939 | 51.97 |
| 14 | wcbH | 1797 | 56.54 |
| 15 | wcbG | 942 | 57.96 |
| 16 | wcbF | 1190 | 54.45 |
| 17 | wcbE | 1525 | 53.57 |
| 18 | wzt2 | 652 | 51.53 |
| 19 | wzm | 783 | 52.11 |
| 20 | wcbD | 1148 | 55.75 |
| 21 | wcbC | 1164 | 59.36 |
| 22 | wcbB | 1152 | 57.81 |
| 23 | wcbA | 2016 | 63.19 |
| 24 | manC | 1431 | 62.68 |
|  | **Average** |  | 59.2 |

References:

1. Yu Y, Kim HS, Chua HH, Lin CH, Sim SH, Lin D, Derr A, Engels R, DeShazer D, Birren B *et al*: **Genomic patterns of pathogen evolution revealed by comparison of Burkholderia pseudomallei, the causative agent of melioidosis, to avirulent Burkholderia thailandensis**. *BMC Microbiol* 2006, **6**:46.

2. Reckseidler SL, DeShazer D, Sokol PA, Woods DE: **Detection of bacterial virulence genes by subtractive hybridization: identification of capsular polysaccharide of Burkholderia pseudomallei as a major virulence determinant**. *Infect Immun* 2001, **69**(1):34-44.
