# Supplementary material for: Genomic acquisition of a capsular polysaccharide virulence cluster by non-pathogenic Burkholderia isolates
Source: Genome Biol. 2010 Aug 27;11(8):R89. doi: 10.1186/gb-2010-11-8-r89 (PMC2945791; doi:10.1186/gb-2010-11-8-r89)
Supplement: Additional file 11 — Experimental validation of 50 representative SNPs predicted in BtE555. [file gb-2010-11-8-r89-S11.DOC]

**Additional data file 11. Sanger Sequencing Validation of BtE555 SNPs.**

|  | **SNP Position** | **SNP ID** | **SNP** | **Sequencing results** |  | **Genotype** | **Conclusion** |
| --- | --- | --- | --- | --- | --- | --- | --- |
|  |  |  |  | **Forward strand** | **Reverse strand** |  |  |
| **1** | 1039 | BTH_CHR1-SNP00003 | G/A | A | A | A | SNP present |
| **2** | 19736 | BTH_CHR1-SNP00063 | T/C | C | C | C | SNP present |
| **3** | 34662 | BTH_CHR1-SNP00123 | A/G | G | G | G | SNP present |
| **4** | 40217 | BTH_CHR1-SNP00142 | T/C | C | C | C | SNP present |
| **5** | 56715 | BTH_CHR1-SNP00218 | A/C | C | C | C | SNP present |
| **6** | 61846 | BTH_CHR1-SNP00247 | A/C | C | C | C | SNP present |
| **7** | 69442 | BTH_CHR1-SNP00285 | C/T | T | T | T | SNP present |
| **8** | 77689 | BTH_CHR1-SNP00332 | G/A | A | A | A | SNP present |
| **9** | 78040 | BTH_CHR1-SNP00337 | C/T | T | T | T | SNP present |
| **10** | 92434 | BTH_CHR1-SNP00405 | T/G | G | G | G | SNP present |
| **11** | 181644 | BTH_CHR1-SNP00553 | C/T | T | T | T | SNP present |
| **12** | 357154 | BTH_CHR1-SNP01276 | A/G | G | G | G | SNP present |
| **13** | 518452 | BTH_CHR1-SNP01923 | T/C | C | C | C | SNP present |
| **14** | 846418 | BTH_CHR1-SNP03322 | T/C | C | C | C | SNP present |
| **15** | 1072470 | BTH_CHR1-SNP04279 | C/T | T | T | T | SNP present |
| **16** | 1088509 | BTH_CHR1-SNP04333 | T/G | G | G | G | SNP present |
| **17** | 1091222 | BTH_CHR1-SNP04348 | G/A | A | A | A | SNP present |
| **18** | 1278419 | BTH_CHR1-SNP05104 | G/A | A | A | A | SNP present |
| **19** | 1610684 | BTH_CHR1-SNP06449 | T/C | C | C | C | SNP present |
| **20** | 1618009 | BTH_CHR1-SNP06498 | A/G | G | G | G | SNP present |
| **21** | 1664882 | BTH_CHR1-SNP06597 | C/T | T | Poor alignment | Possibly T | Undetermined |
| **22** | 2085057 | BTH_CHR1-SNP08463 | G/A | Poor alignment | Poor alignment | Undetermined | Undetermined |
| **23** | 2193766 | BTH_CHR1-SNP08926 | T/C | C | C | C | SNP present |
| **24** | 2700855 | BTH_CHR1-SNP11274 | G/A | A | A | A | SNP present |
| **25** | 3484038 | BTH_CHR1-SNP14580 | G/A | A | A | A | SNP present |
| **26** | 3807495 | BTH_CHR1-SNP15836 | A/G | G | G | G | SNP present |
| **27** | 14382 | BTH_CHR2-SNP15926 | C/T | T | T | T | SNP present |
| **28** | 16566 | BTH_CHR2-SNP15939 | A/G | G | G | G | SNP present |
| **29** | 32568 | BTH_CHR2-SNP16014 | G/A | A | A | A | SNP present |
| **30** | 67549 | BTH_CHR2-SNP16202 | C/T | T | T | T | SNP present |
| **31** | 70774 | BTH_CHR2-SNP16241 | G/T | T | T | T | SNP present |
| **32** | 71391 | BTH_CHR2-SNP16245 | C/T | No PCR | No PCR | Undetermined | Undetermined |
| **33** | 76983 | BTH_CHR2-SNP16298 | A/C | C | C | C | SNP present |
| **34** | 124115 | BTH_CHR2-SNP16446 | A/C | C | C | C | SNP present |
| **35** | 124938 | BTH_CHR2-SNP16453 | A/G | G | G | G | SNP present |
| **36** | 130291 | BTH_CHR2-SNP16501 | C/T | T | T | T | SNP present |
| **37** | 133290 | BTH_CHR2-SNP16525 | T/C | C | C | C | SNP present |
| **38** | 137312 | BTH_CHR2-SNP16544 | T/C | C | C | C | SNP present |
| **39** | 139404 | BTH_CHR2-SNP16561 | T/C | C | C | C | SNP present |
| **40** | 1892512 | BTH_CHR2-SNP26113 | G/T | T | T | T | SNP present |
| **41** | 2060147 | BTH_CHR2-SNP26853 | T/C | C | C | C | SNP present |
| **42** | 2062530 | BTH_CHR2-SNP26871 | C/T | T | T | T | SNP present |
| **43** | 2068619 | BTH_CHR2-SNP26904 | A/G | G | G | G | SNP present |
| **44** | 2094988 | BTH_CHR2-SNP27034 | G/A | A | A | A | SNP present |
| **45** | 2128766 | BTH_CHR2-SNP27221 | T/C | C | C | C | SNP present |
| **46** | 2147143 | BTH_CHR2-SNP27339 | G/A | A | A | A | SNP present |
| **47** | 2156657 | BTH_CHR2-SNP27379 | A/G | G | G | G | SNP present |
| **48** | 2156884 | BTH_CHR2-SNP27380 | C/T | T | T | T | SNP present |
| **49** | 2240280 | BTH_CHR2-SNP27814 | C/T | T | T | T | SNP present |
| **50** | 2906326 | BTH_CHR2-SNP30925 | C/T | T | T | T | SNP present |

**Additional data file 11. Sanger Sequencing Validation of BtE555 SNPs.**

50 randomly selected SNPs inferred from analysis of the BtE555 and BtE264 genome sequences were chosen for validation. Only 3 out of 50 SNPs could not be successfully validated, largely due to poor sequence quality.
