# Supplementary material for: Genomic acquisition of a capsular polysaccharide virulence cluster by non-pathogenic Burkholderia isolates
Source: Genome Biol. 2010 Aug 27;11(8):R89. doi: 10.1186/gb-2010-11-8-r89 (PMC2945791; doi:10.1186/gb-2010-11-8-r89)
Supplement: Additional file 12 — Sequence statistics of the de novo assembled contigs from BtE555 deep sequencing paired-end reads. [file gb-2010-11-8-r89-S12.DOC]

**Additional data file 12. Assembly Statistics.**

| Assembly  Parameters | Statistics |
| --- | --- |
| No. of contigs | 521 |
| Total length | 6145909 bp |
| Average length | 11796 bp |
| Max length | 72827 bp |
| Min length | 250 bp |
| N50 length | 20293 bp |

**Additional data file 12. Assembly Statistics.**

Sequence statistics of *de novo* assembled contigs from BtE555 deep sequencing paired-end reads.
