# Supplementary material for: Genomic acquisition of a capsular polysaccharide virulence cluster by non-pathogenic Burkholderia isolates
Source: Genome Biol. 2010 Aug 27;11(8):R89. doi: 10.1186/gb-2010-11-8-r89 (PMC2945791; doi:10.1186/gb-2010-11-8-r89)
Supplement: Additional file 13 — A table that ascribes BtE555 contigs that are mappable to the GI, nGi and EPS regions in BtE264. [file gb-2010-11-8-r89-S13.DOC]

**Additional data file 13. BtE555 contigs mapping to GI/nGi/EPS regions in Bt E264**

| **S.No** | **Bt E264** | **Contig ID** | **Length of contig** | **5' contig co-ordinates** | **Bt E264 genomic co-ordinate** | **3' contig co-ordinates** | **Bt E264 genomic co-ordinate** | **Size in BtE555**  **(bp)** | **Size in BtE264**  **(bp)** |
| --- | --- | --- | --- | --- | --- | --- | --- | --- | --- |
| **1** | nGi02 | 256 | 7829 | 1..3908 | (555173..559086) | 5962..7829 | (562353..564220) | 2053 | 3266 |
| **2** | nGi03 | 256 | 7829 | 1..3908 | (555173..559086) | 5962..7829 | (562353..564220) | 2053 | 3266 |
| **3** | nGi04 | 9 | 30617 | 24..5382 | (729343..734713) | 16964..30617 | (736132..749777) | 11581 | 1418 |
| **4** | nGi05^*^ | 138 | 8635 | 46..5301 | (838030..843297) | 5292..8635 | (844601..847944) | - | - |
| **5** | YT02 | 33 | 41003 | 2437..1 | (1066739..1069189) | 41003..29068 | c(1025512..1037394) | 26630 | 29344 |
| **6** | EPS | 19 | 38859 | 2243, 332 | (1516939..1518832) | 38859.. 34097 | c(1482208..1486970) | 31853 | 29968 |
| **7** | YT03 | 16 | 36337 | 1..994 | (1624419..1625412) | 1258..36337 | (1653202..1688290) | 263 | 27789 |
| **8** | nGi07 | 31 | 50424 | 1..9071 | (1703280..1712350) | 39831..50424 | (1749682..1760277) | 30759 | 37331 |
| **9** | nGi08 | 31 | 50424 | 1..9071 | (1703280..1712350) | 39831..50424 | (1749682..1760277) | 30759 | 37331 |
| **10** | nGi09 | 95 | 41796 | 1..19218 | (1932461..1951678) | 29241..41794 | (1964844..1977371) | 10022 | 13165 |
| **11** | YT04 | 32 | 51846 | 12433..1 | (2193669..2206094) | 51846..28021 | c(2138453..2162276) | 15587 | 31392 |
| **12** | nGi11 | 239 | 30489 | 1..1423 | (2933893..2935315) | 11927..30489 | (2948395..2966947) | 10503 | 13079 |
| **13** | YT05 | 64 | 51405 | 1..10772 | (3101285..3112057) | 24990..51405 | (3173947..3200343) | 14217 | 61889 |
| **14** | nGi14 | 64 | 51405 | 1..10772 | (3101285..3112057) | 24990..51405 | (3173947..3200343) | 14217 | 61889 |
| **15** | nGi15 | 195 | 17102 | 4558..1 | (3254846..3259403) | 17102..4562 | c(3240913..3253394) | 3 | 1451 |
| **16** | YT07 | 243 | 45739 | 53..15056 | (3401386..3416397) | 23768..45739 | (3417517..3439485) | 8711 | 1119 |
| **17** | nGi16 | 275 | 16469 | 1..10372 | (3629793..3640145) | 10373..16466 | (3641596..3647689) | - | - |
| **18** | YT11 | 248 | 43735 | 1..7301 | (418627..425924) | 20127..43690 | (441039..464582) | 12825 | 15114 |
| **19** | YT13 | 186 | 26099 | 17979..1 | (1614932..1632909) | 26099..18007 | c(1570610..1578689) | 27 | 36242 |
| **20** | nGi23 | 328 | 3189 | 1..1845 | (1813345..1815192) | 1853..3189 | (1816670..1818006) | 7 | 1477 |
| **21** | nGi24 | 142 | 6261 | 4248..1 | (1943836..1948083) | 6261..4248 | c(1940497..1942523) | - | - |
| **22** | nGi25^*^ | 439 | 6909 | 6909..1 | (2734288..2741195) | 6909..1 | c(2734288..2741195) | 6909 | 6906 |
| **23** | nGi26^*^ | 439 | 6909 | 6909..1 | (2734288..2741195) | 6909..1 | c(2734288..2741195) | 6909 | 6906 |

**Col2:** GI/nGi/EPS elements in BtE264 identified by computational analysis or aCGH

**Col3:** BtE555 contig identifier mapped to the BtE264 genomic element

**Col4:** Length of contig in base pairs (bp)

**Col5&7:** Contig co-ordinates mapping to the 5’ and 3’ flanking region of the genomic element, conserved in both strains

**Col 6&8:** Corresponding genomic co-ordinates in BtE264

**Col9:** Length of the BtE555-specific region in the BtE555 contig (in bp).

**Col10**: Length of the BtE264 region for which there is no sequence similarity within the BtE555 contig (in bp)
