# Supplementary material for: Genomic acquisition of a capsular polysaccharide virulence cluster by non-pathogenic Burkholderia isolates
Source: Genome Biol. 2010 Aug 27;11(8):R89. doi: 10.1186/gb-2010-11-8-r89 (PMC2945791; doi:10.1186/gb-2010-11-8-r89)
Supplement: Additional file 14 — A list of the BtE555 unique genes and their associated contigs, when compared against reference strain BtE264. [file gb-2010-11-8-r89-S14.DOC]

**Additional data file 14. BtE555 specific genes inferred from contig sequence**

| **Contig ID** | **No. of Bt E555 specific genes** | **Functional annotation** |
| --- | --- | --- |
| 0 | 7 | gp30; Putative bacteriophage gp30 protein; Uncharacterized conserved protein; |
| 5 | 9 | Hypothetical protein ; Hypothetical protein ; |
| 9 | 14 | Integrase; Type II secretory pathway, component PulD; hypothetical protein; |
| 14 | 3 | Permeases of the major facilitator superfamily; Metal-dependent amidase/aminoacylase/carboxypeptidase; Transcriptional regulator; |
| 19 | 21 | Polyketide synthase modules and related proteins; Phosphoglycerol transferase and related proteins, alkaline phosphatase superfamily; Dehydrogenases with different specificities (related to short-chain alcohol dehydrogenases); Capsule polysaccharide export protein; Histidinol phosphatase and related phosphatases; Nucleoside-diphosphate-sugar pyrophosphorylase involved in lipopolysaccharide biosynthesis/translation initiation factor 2B, gamma/epsilon subunits (eIF-2Bgamma/eIF-2Bepsilon); Phosphoheptose isomerase; Predicted kinase related to galactokinase and mevalonate kinase; Nucleoside-diphosphate-sugar epimerases; WcbJ ; Putative capsular polysaccharide biosynthesis; Glycosyltransferase; WcbG ; Hypothetical protein ; Glycosyltransferase; ABC-type polysaccharide/polyol phosphate transport system, ATPase component; ABC-type polysaccharide/polyol phosphate export systems, permease component; Capsule polysaccharide export protein; Periplasmic protein involved in polysaccharide export; Glycosyltransferase; Capsule polysaccharide export protein; |
| 20 | 16 | Uncharacterized conserved protein; Putative multicopper oxidases; Putative IS element transposase; Cyclopropane fatty acid synthase and related methyltransferases; Uncharacterized conserved protein; Predicted NAD/FAD-binding protein; Bacterial lipocalin; Cyclopropane fatty acid synthase and related methyltransferases; Uncharacterized protein conserved in bacteria; DNA-directed RNA polymerase specialized sigma subunit, sigma24 homolog; Bacterial lipocalin; Uncharacterized protein conserved in bacteria; NADH:ubiquinone oxidoreductase subunit 5 (chain L)/Multisubunit Na+/H+ antiporter, MnhA subunit; Transcriptional regulator; |
| 21 | 6 | hypothetical protein; Putative bacteriophage coat protein; Putative membrane protein; Putative membrane protein; |
| 24 | 3 | Transposase and inactivated derivatives; |
| 28 | 29 | phage major capsid protein, HK97 family; Phage head maturation protease; Phage-related protein; Phage terminase-like protein, large subunit; phage terminase, small subunit, putative, P27; Cytotoxic translational repressor of; Predicted transcriptional regulators; Predicted ATP-binding protein involved in virulence; Site-specific DNA methylase; Gp46; Bacteriophage protein gp37; Hypothetical protein; Integrase; |
| 29 | 8 | Putative bacteriophage gp30 protein; gp30; |
| 31 | 2 | transcriptional regulator, LysR family; Dehydrogenases with different specificities (related to short-chain alcohol dehydrogenases); |
| 33 | 23 | Integrase; Hypothetical protein ; Hypothetical protein ; Putative phage-related protein; Phage terminase-like protein, large subunit; Phage head maturation protease; Phage-related protein; Hypothetical protein ; hypothetical protein; hypothetical protein; |
| 36 | 42 | Growth inhibitor; Predicted transcriptional regulator; Predicted P-loop ATPase and inactivated derivatives; Bacteriophage tail assembly protein; Hypothetical protein ; Bacteriophage capsid protein; Protease subunit of ATP-dependent Clp proteases; Hypothetical protein ; Hypothetical protein; Hypothetical protein; Phage P2 baseplate assembly protein gpV; Phage baseplate assembly protein W; Phage-related baseplate assembly protein; Bacteriophage P2-related tail formation protein; Phage-related tail fibre protein; Putative phage tail fibre protein; Phage tail sheath protein FI; Phage tail tube protein FII; Hypothetical protein ; Mu-like prophage protein; |
| 48 | 4 | Putative hemagglutinin/hemolysin- related; Hypothetical protein ; hypothetical protein; |
| 54 | 5 | GGDEF domain protein; Glycosyltransferases, probably involved in cell wall biogenesis; Glycosyltransferases, probably involved in cell wall biogenesis; |
| 55 | 13 | transcriptional regulator, MerR family; Hypothetical protein ; Arabinose efflux permease; Putative translation initiation inhibitor, yjgF family; hypothetical protein; Predicted transcriptional regulators; Hypothetical protein ; |
| 57 | 5 | Sll1273 protein ; Plasmid stabilization system protein; hypothetical protein; Integrase; |
| 58 | 4 | Type II secretory pathway, component PulD; Zonula occludens toxin; Putative membrane protein; |
| 60 | 21 | Hypothetical protein ; hypothetical protein; Conserved hypothetical phage-related protein; Type II secretory pathway, component ExeA (predicted ATPase); Hypothetical protein ; Uncharacterized conserved protein; hypothetical protein; TniB; Integrase, catalytic region; ATP-dependent endonuclease of the OLD; DNA-binding protein H-NS; Superfamily I DNA and RNA helicases and helicase subunits; Putative phosphoesterase; |
| 72 | 7 | Putative phage tail fiber assembly protein; Phage-related tail fibre protein; Bacteriophage P2-related tail formation protein; Phage-related baseplate assembly protein; Phage baseplate assembly protein W; hypothetical protein; Putative transposase; |
| 73 | 3 | Transposase and inactivated derivatives; Transposase and inactivated derivatives; Transposase and inactivated derivatives; |
| 77 | 7 | DNA replication protein; Predicted transcriptional regulator; Uncharacterized protein conserved in bacteria; DNA G:T-mismatch repair endonuclease; Site-specific DNA methylase; |
| 78 | 5 | Transcriptional regulator; Thioredoxin reductase; SAM-dependent methyltransferases; hypothetical protein; |
| 79 | 2 | Autotransporter adhesin; |
| 102 | 7 | PEP phosphonomutase and related enzymes; Thiamine pyrophosphate-requiring enzymes [acetolactate synthase, pyruvate dehydrogenase (cytochrome), glyoxylate carboligase, phosphonopyruvate decarboxylase]; Probable taurine catabolism dioxygenase; NAD-dependent aldehyde dehydrogenases; Transcriptional regulator; Predicted permeases; |
| 124 | 5 | Signal transduction histidine kinase; Response regulators consisting of a CheY-like receiver domain and a winged-helix DNA-binding domain; Outer membrane protein (porin); Outer membrane protein (porin); |
| 156 | 11 | AraC-type DNA-binding domain-containing proteins; Short-chain dehydrogenases of various substrate specificities; NADH:flavin oxidoreductases, Old Yellow Enzyme family; Predicted metal-dependent hydrolase; AraC-type DNA-binding domain-containing proteins; Choline dehydrogenase and related flavoproteins; Predicted flavoprotein involved in K+ transport; hypothetical protein; Alpha/beta hydrolase; |
| 212 | 1 | Transposase and inactivated derivatives; |
| 220 | 2 | Transposase and inactivated derivatives; |
| 233 | 18 | DNA-binding HTH domain-containing proteins; N-acyl-L-homoserine lactone synthetase; Non-ribosomal peptide synthetase modules and related proteins; Asp-tRNAAsn/Glu-tRNAGln amidotransferase A subunit and related amidases; Permeases of the major facilitator superfamily; Aspartyl-tRNA synthetase; Predicted pyridoxal phosphate-dependent enzyme apparently involved in regulation of cell wall biogenesis; 3-dehydroquinate synthetase; Xylose isomerase-like TIM barrel; Hypothetical protein ; Dehydrogenases with different specificities (related to short-chain alcohol dehydrogenases); Transposase and inactivated derivatives; Transposase and inactivated derivatives; Transposase and inactivated derivatives; |
| 235 | 3 | Amidases related to nicotinamidase; Permeases of the major facilitator superfamily; Transcriptional regulators; |
| 237 | 2 | putative non-ribosomal peptide synthetase; Hypothetical protein ; |
| 238 | 5 | ATPases involved in chromosome partitioning; |
| 242 | 6 | Response regulator containing a CheY-like receiver domain and an HTH DNA-binding domain; Signal transduction histidine kinase; Response regulators consisting of a CheY-like receiver domain and a winged-helix DNA-binding domain; |
| 243 | 12 | Integrase; Uncharacterized protein conserved in bacteria; Predicted transcriptional regulator; plasmid related protein; plasmid related protein; Predicted transcriptional regulators; Transcriptional regulator, GntR family; |
| 247 | 3 | Superfamily II helicase and inactivated derivatives; |
| 248 | 6 | Phenylpropionate dioxygenase and related ring-hydroxylating dioxygenases, large terminal subunit; PrnC; hypothetical protein; tryptophan halogenase; Transposase and inactivated derivatives; |
| 256 | 3 |  |
| 262 | 5 | Type I site-specific restriction-modification system, R (restriction) subunit and related helicases; Hypothetical protein ; |
| 277 | 2 | hypothetical protein; lipoprotein, putative; |
| 282 | 2 | Phenylpropionate dioxygenase and related ring-hydroxylating dioxygenases, large terminal subunit; |
| 284 | 2 | Transposase and inactivated derivatives; Integrase; |
| 288 | 21 | Predicted transcriptional regulators; Putative phage DNA-binding protein; Putative phage protein ; Prophage antirepressor; Conserved hypothetical phage protein; Integrase; Predicted Zn peptidase; Integrase; |
| 303 | 1 | Site-specific recombinases, DNA invertase Pin homologs; |
| 317 | 4 | Outer membrane protein; Membrane-fusion protein; Cation/multidrug efflux pump; Response regulators consisting of a CheY-like receiver domain and a winged-helix DNA-binding domain; |
| 320 | 1 | Integrase; |
| 325 | 1 | Transcriptional regulators containing a DNA-binding HTH domain and an aminotransferase domain (MocR family) and their eukaryotic orthologs; |
| 397 | 2 | D-alanyl-D-alanine carboxypeptidase; Cell division protein FtsI/penicillin-binding protein 2; |
| 402 | 4 | hypothetical protein; hypothetical protein; Hypothetical protein ; Hypothetical protein ; |
| 403 | 6 | ABC-type transport system, involved in lipoprotein release, permease component; protein of unknown function DUF214; Citrate synthase; |
| 412 | 2 | Mu-like prophage protein; Phage protein U; |
| 442 | 3 | Transcriptional regulator; Beta-lactamase class C and other penicillin binding proteins; Arabinose efflux permease; |
