# Supplementary material for: Genomic acquisition of a capsular polysaccharide virulence cluster by non-pathogenic Burkholderia isolates
Source: Genome Biol. 2010 Aug 27;11(8):R89. doi: 10.1186/gb-2010-11-8-r89 (PMC2945791; doi:10.1186/gb-2010-11-8-r89)
Supplement: Additional file 15 — Two phylogenetic trees drawn with permuted data, to ensure that the aCGH clusters are robust. [file gb-2010-11-8-r89-S15.DOC]

**Additional data file 15.** **Robustness of aCGH clustering.**


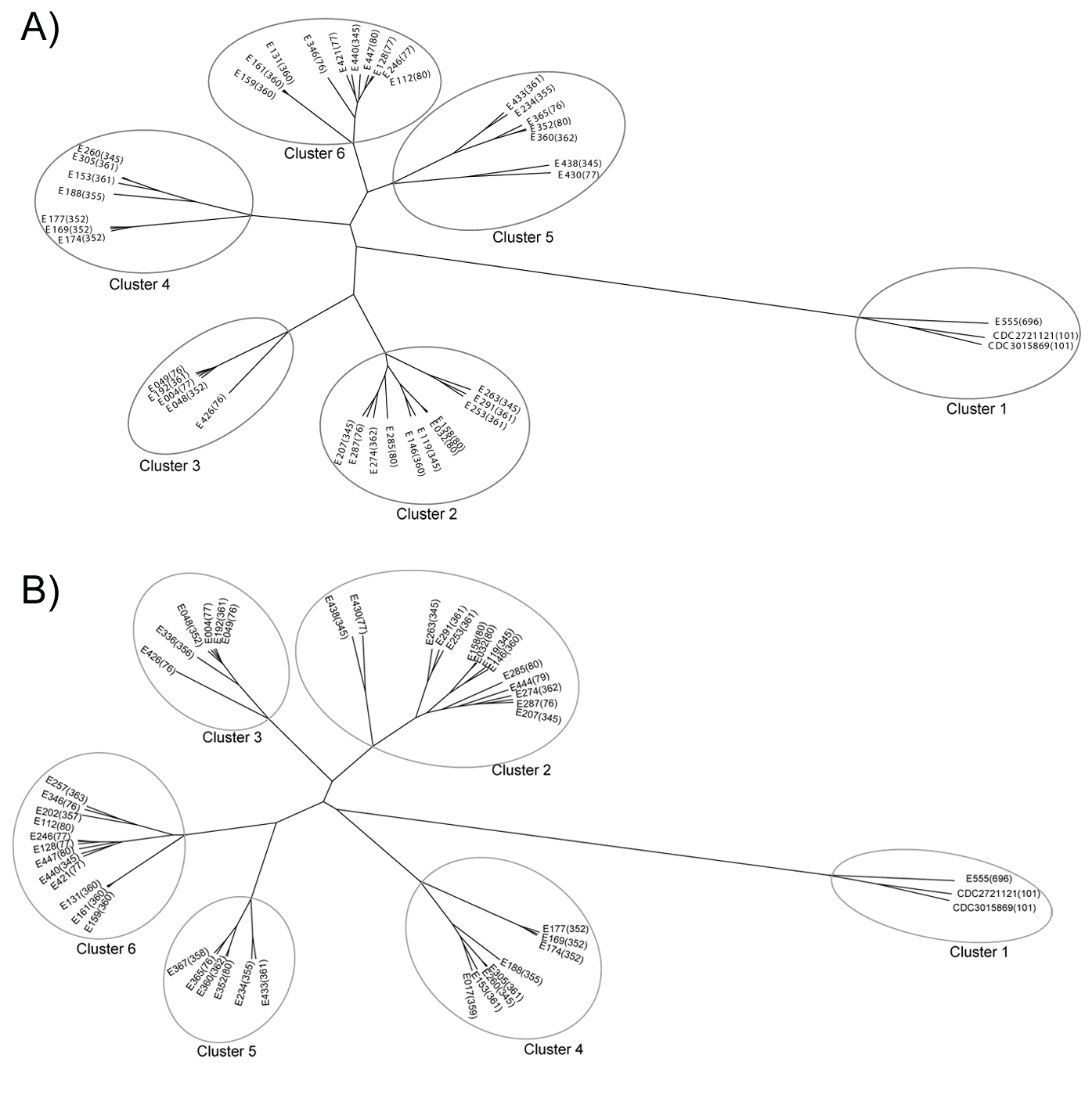


**Additional data file 15.** **Robustness of aCGH clustering.** The robustness of the phylogenetic tree generated by aCGH clustering was assessed using two methods. (A) Removal of ST singletons. All STs represented by a single strain were excluded from clustering (6 strains excluding BtE555). (B) Data removal. A random 15% of the BtE264 microarray probes were removed from analysis, and the strains reclustered. This process was iterated 100 times. In both A) and B), six distinct clusters were observed with near-identical strain compositions (only two strains, E430 and E438 appear unstable). Cluster 1, the outlier subgroup, appears distinct in both A) and B).
