# Supplementary material for: Genomic acquisition of a capsular polysaccharide virulence cluster by non-pathogenic Burkholderia isolates
Source: Genome Biol. 2010 Aug 27;11(8):R89. doi: 10.1186/gb-2010-11-8-r89 (PMC2945791; doi:10.1186/gb-2010-11-8-r89)
Supplement: Additional file 16 — List of genes absent in three of the strains in cluster 1 (which contains the variant strains) when compared against BtE264. [file gb-2010-11-8-r89-S16.DOC]

**Additional data file 16. Genes absent in Cluster 1 strains compared to Bt E264.**

| CHR | Gene | Number of probes lost in strains in C1 | Total number of probes that covers the gene | % Lost | Gene start | Gene stop |
| --- | --- | --- | --- | --- | --- | --- |
| 1 | hypothetical protein BTH_I0077 | 4 | 4 | 100.00 | 83375 | 83782 |
| 1 | transposase | 1 | 22 | 4.55 | 559553 | 561472 |
| 1 | TrapT dctQ-M fusion permease, dicarboxylate transport | 20 | 28 | 71.43 | 921969 | 923648 |
| 1 | TRAP dicarboxylate family transporter DctP subunit | 18 | 18 | 100.00 | 923691 | 924701 |
| 1 | ribose operon repressor, putative | 12 | 18 | 66.67 | 924817 | 925848 |
| 1 | transposase mutator family protein | 16 | 22 | 72.73 | 1047571 | 1048830 |
| 1 | hypothetical protein BTH_I1101 | 5 | 5 | 100.00 | 1242993 | 1243400 |
| 1 | TnpB protein | 7 | 7 | 100.00 | 1243397 | 1243783 |
| 1 | transposase mutator family protein | 21 | 21 | 100.00 | 1243753 | 1245012 |
| 1 | TnpC protein | 7 | 7 | 100.00 | 1245083 | 1245658 |
| 1 | TnpC protein | 24 | 24 | 100.00 | 1625456 | 1627027 |
| 1 | TnpB protein | 6 | 6 | 100.00 | 1627057 | 1627404 |
| 1 | hypothetical protein BTH_I1441 | 4 | 4 | 100.00 | 1627401 | 1627808 |
| 1 | hypothetical protein BTH_I1445 | 2 | 4 | 50.00 | 1637392 | 1637799 |
| 1 | TnpB protein | 6 | 6 | 100.00 | 1637796 | 1638143 |
| 1 | TnpC protein | 20 | 23 | 86.96 | 1638173 | 1639744 |
| 1 | TnpB protein | 7 | 7 | 100.00 | 1639848 | 1640195 |
| 1 | TnpC protein | 24 | 24 | 100.00 | 1640225 | 1641796 |
| 1 | TnpC protein | 19 | 25 | 76.00 | 1642905 | 1644476 |
| 1 | TnpB protein | 5 | 6 | 83.33 | 1644506 | 1644853 |
| 1 | hypothetical protein BTH_I1452 | 4 | 4 | 100.00 | 1644850 | 1645257 |
| 1 | hypothetical protein BTH_I1532 | 17 | 17 | 100.00 | 1734399 | 1735379 |
| 1 | Doc protein | 8 | 8 | 100.00 | 1740773 | 1741156 |
| 1 | riboflavin synthase subunit alpha | 2 | 12 | 16.67 | 1741200 | 1741829 |
| 1 | hypothetical protein BTH_I2330 | 1 | 18 | 5.56 | 2622323 | 2623372 |
| 1 | Rieske (2Fe-2S) domain-containing protein | 2 | 18 | 11.11 | 2623372 | 2624352 |
| 1 | FAD binding domain-containing protein | 10 | 25 | 40.00 | 2625779 | 2627221 |
| 1 | hypothetical protein BTH_I2334 | 12 | 12 | 100.00 | 2627362 | 2628060 |
| 1 | Sea27 | 3 | 11 | 27.27 | 2649156 | 2649734 |
| 1 | arachidonate 15-lipoxygenase precursor (15-LOX) | 29 | 35 | 82.86 | 2650433 | 2652520 |
| 1 | hypothetical protein BTH_I2354 | 20 | 20 | 100.00 | 2652517 | 2653629 |
| 1 | alpha/beta fold family hydrolase | 17 | 17 | 100.00 | 2653679 | 2654716 |
| 1 | serine protease | 1 | 10 | 10.00 | 2654881 | 2655453 |
| 1 | TnpC protein | 23 | 23 | 100.00 | 3070382 | 3071953 |
| 1 | TnpB protein | 6 | 6 | 100.00 | 3071983 | 3072330 |
| 1 | hypothetical protein BTH_I2690 | 4 | 4 | 100.00 | 3072327 | 3072734 |
| 1 | TnpC protein | 23 | 23 | 100.00 | 3141963 | 3143534 |
| 1 | TnpB protein | 6 | 6 | 100.00 | 3143564 | 3143911 |
| 1 | hypothetical protein BTH_I2737 | 3 | 4 | 75.00 | 3143908 | 3144315 |
| 1 | transposase mutator family protein | 12 | 21 | 57.14 | 3158707 | 3159966 |
| 1 | hypothetical protein BTH_I2889 | 4 | 4 | 100.00 | 3325125 | 3325532 |
| 1 | TnpB protein | 6 | 6 | 100.00 | 3325529 | 3325855 |
| 1 | TnpC protein | 9 | 9 | 100.00 | 3325759 | 3326226 |
| 1 | TnpC protein | 2 | 2 | 100.00 | 3327577 | 3327804 |
| 1 | hypothetical protein BTH_I3089 | 4 | 4 | 100.00 | 3523544 | 3523951 |
| 1 | TnpB protein | 6 | 6 | 100.00 | 3523948 | 3524274 |
| 1 | transposase mutator family protein | 10 | 21 | 47.62 | 3568217 | 3569476 |
| 1 | phage integrase family site specific recombinase | 1 | 13 | 7.69 | 3569536 | 3570204 |
| 1 | TnpC protein | 14 | 23 | 60.87 | 3583408 | 3584979 |
| 1 | TnpB protein | 5 | 6 | 83.33 | 3585009 | 3585356 |
| 1 | hypothetical protein BTH_I3142 | 4 | 4 | 100.00 | 3585353 | 3585760 |
| 1 | DNA-binding protein BprA | 5 | 5 | 100.00 | 3670691 | 3670996 |
| 1 | Rhs element Vgr protein, putative | 23 | 47 | 48.94 | 3671318 | 3674098 |
| 1 | hypothetical protein BTH_I3226 | 24 | 37 | 64.86 | 3674166 | 3676352 |
| 1 | hypothetical protein BTH_I3227 | 10 | 27 | 37.04 | 3676491 | 3678029 |
| 1 | PAAR motif-containing protein | 4 | 5 | 80.00 | 3678039 | 3678302 |
| 1 | transposase mutator family protein | 7 | 21 | 33.33 | 3734306 | 3735496 |
| 2 | K+-transporting ATPase, A subunit | 4 | 11 | 36.36 | 6192 | 6740 |
| 2 | IS407A, transposase OrfA | 1 | 6 | 16.67 | 88930 | 89187 |
| 2 | transposase subunit | 3 | 3 | 100.00 | 89295 | 89432 |
| 2 | transposase subunit | 6 | 6 | 100.00 | 89575 | 89862 |
| 2 | acetyltransferase | 4 | 9 | 44.44 | 91572 | 92024 |
| 2 | putrescine ABC transporter, permease protein | 5 | 13 | 38.46 | 92677 | 93405 |
| 2 | ABC transporter, permease protein | 9 | 15 | 60.00 | 93392 | 94246 |
| 2 | putrescine ABC transporter, periplasmic putrescine-binding protein, putative | 7 | 18 | 38.89 | 94243 | 95274 |
| 2 | transposase | 1 | 19 | 5.26 | 95823 | 96893 |
| 2 | DNA-binding protein BprA | 3 | 6 | 50.00 | 97053 | 97358 |
| 2 | Rhs element Vgr protein, putative | 14 | 48 | 29.17 | 97680 | 100460 |
| 2 | hypothetical protein BTH_II0090 | 9 | 37 | 24.32 | 100528 | 102714 |
| 2 | hypothetical protein BTH_II0091 | 3 | 26 | 11.54 | 102853 | 104391 |
| 2 | PAAR motif-containing protein | 3 | 6 | 50.00 | 108712 | 108975 |
| 2 | short chain dehydrogenase/reductase family oxidoreductase | 7 | 14 | 50.00 | 426274 | 427047 |
| 2 | LysR family transcriptional regulator | 8 | 16 | 50.00 | 427198 | 428085 |
| 2 | hypothetical protein BTH_II0351 | 2 | 4 | 50.00 | 428174 | 428308 |
| 2 | thermoresistant gluconokinase | 4 | 9 | 44.44 | 428980 | 429486 |
| 2 | major facilitator family transporter | 12 | 23 | 52.17 | 429534 | 430832 |
| 2 | transposase mutator family protein | 13 | 21 | 61.90 | 432555 | 433814 |
| 2 | transposase mutator family protein | 13 | 21 | 61.90 | 515757 | 517016 |
| 2 | lectin repeat-containing protein | 19 | 42 | 45.24 | 858567 | 860987 |
| 2 | NAD synthetase | 4 | 40 | 10.00 | 1078629 | 1081037 |
| 2 | TnpC protein | 4 | 4 | 100.00 | 2490052 | 2490426 |
| 2 | hypothetical protein BTH_II2075 | 5 | 5 | 100.00 | 2530743 | 2531054 |
| 2 | hypothetical protein BTH_II2076 | 1 | 3 | 33.33 | 2531437 | 2531559 |
| 2 | Rieske (2Fe-2S) domain-containing protein | 1 | 18 | 5.56 | 2606460 | 2607452 |
| 2 | O-methyltransferase family protein | 8 | 21 | 38.10 | 2608589 | 2609704 |

**Additional data file 16. Genes absent in Cluster 1 strains compared to BtE264.**

Listed are the BtE264 genes predicted to be absent in all three cluster 1 strains (BtE555, BtCDC3015869, and Bt CDC2721121) by aCGH analysis. Many of the genes are related to recombination (eg transposases and integrases), permeases/transporters, and DNA binding proteins.
