# Supplementary material for: Genomic acquisition of a capsular polysaccharide virulence cluster by non-pathogenic Burkholderia isolates
Source: Genome Biol. 2010 Aug 27;11(8):R89. doi: 10.1186/gb-2010-11-8-r89 (PMC2945791; doi:10.1186/gb-2010-11-8-r89)
Supplement: Additional file 17 — A list of strains used for both MLST and aCGH analysis. [file gb-2010-11-8-r89-S17.DOC]

**Additional data file 17. List of Bt Strains used for MLST Analysis**

| No.  aCGH | No. MLST | Species | Strain ID | Year of isolation | Source | Country of origin | ST | MLST |
| --- | --- | --- | --- | --- | --- | --- | --- | --- |
| 1 | 1 | *B. thailandensis* | E0049 | 1992 | Soil | Thailand | 76 | This study |
|  | 2 | *B. thailandensis* | E0255 | 1992 | Soil | Thailand | 76 | This study |
| 2 | 3 | *B. thailandensis* | E0287 | 1994 | Soil | Thailand | 76 | This study |
|  | 4 | *B. thailandensis* | E0290 | 1994 | Soil | Thailand | 76 | This study |
|  | 5 | *B. thailandensis* | E0299 | 1994 | Soil | Thailand | 76 | This study |
| 3 | 6 | *B. thailandensis* | E0346 | 1998 | Soil | Thailand | 76 | This study |
|  | 7 | *B. thailandensis* | E0354 | 1998 | Soil | Thailand | 76 | This study |
|  | 8 | *B. thailandensis* | E0364 | 1998 | Soil | Thailand | 76 | This study |
| 4 | 9 | *B. thailandensis* | E0365 | 1998 | Soil | Thailand | 76 | This study |
| 5 | 10 | *B. thailandensis* | E0426 | 2001 | Soil | Thailand | 76 | This study |
| 6 | 11 | *B. thailandensis* | E0004 | 1990 | Soil | Thailand | 77 | This study |
|  | 12 | *B. thailandensis* | E0005 | 1990 | Soil | Thailand | 77 | This study |
|  | 13 | *B. thailandensis* | E0028 | 1990 | Soil | Thailand | 77 | This study |
| 7 | 14 | *B. thailandensis* | E0128 | 1991 | Soil | Thailand | 77 | This study |
|  | 15 | *B. thailandensis* | E0205 | 1992 | Soil | Thailand | 77 | This study |
|  | 16 | *B. thailandensis* | E0229 | 1992 | Soil | Thailand | 77 | This study |
| 8 | 17 | *B. thailandensis* | E0246 | 1992 | Soil | Thailand | 77 | This study |
| 9 | 18 | *B. thailandensis* | E0421 | 2001 | Soil | Thailand | 77 | This study |
| 10 | 19 | *B. thailandensis* | E0430 | 2002 | Soil | Thailand | 77 | This study |
| 11 | 20 | *B. thailandensis* | E0444 | 2002 | Soil | Thailand | 79 | This study |
| 12 | 21 | *B. thailandensis* | E0032 | 1990 | Soil | Thailand | 80 | This study |
|  | 22 | *B. thailandensis* | E0042 | 1990 | Soil | Thailand | 80 | This study |
| 13 | 23 | *B. thailandensis* | E0112 | 1991 | Soil | Thailand | 80 | This study |
|  | 24 | *B. thailandensis* | E0121 | 1991 | Soil | Thailand | 80 | This study |
|  | 25 | *B. thailandensis* | E0122 | 1991 | Soil | Thailand | 80 | This study |
|  | 26 | *B. thailandensis* | E0123 | 1991 | Soil | Thailand | 80 | This study |
|  | 27 | *B. thailandensis* | E0151 | 1991 | Soil | Thailand | 80 | This study |
|  | 28 | *B. thailandensis* | E0152 | 1991 | Soil | Thailand | 80 | This study |
|  | 29 | *B. thailandensis* | E0154 | 1991 | Soil | Thailand | 80 | This study |
| 14 | 30 | *B. thailandensis* | E0158 | 1991 | Soil | Thailand | 80 | This study |
|  | 31 | *B. thailandensis* | E0201 | 1992 | Soil | Thailand | 80 | This study |
| 15 | 32 | *B. thailandensis* | E0285 | 1993 | Soil | Thailand | 80 | This study |
| 16 | 33 | *B. thailandensis* | E0352 | 1998 | Soil | Thailand | 80 | This study |
|  | 34 | *B. thailandensis* | E0427 | 2002 | Soil | Thailand | 80 | This study |
|  | 35 | *B. thailandensis* | E0435 | 2002 | Soil | Thailand | 80 | This study |
| 17 | 36 | *B. thailandensis* | E0447 | 2002 | Soil | Thailand | 80 | This study |
| 18 | 37 | *B. thailandensis* | E0119 | 1991 | Soil | Thailand | 345 | This study |
|  | 38 | *B. thailandensis* | E0148 | 1991 | Soil | Thailand | 345 | This study |
|  | 39 | *B. thailandensis* | E0149 | 1991 | Soil | Thailand | 345 | This study |
|  | 40 | *B. thailandensis* | E0175 | 1991 | Soil | Thailand | 345 | This study |
| 19 | 41 | *B. thailandensis* | E0207 | 1992 | Soil | Thailand | 345 | This study |
|  | 42 | *B. thailandensis* | E0232 | 1992 | Soil | Thailand | 345 | This study |
|  | 43 | *B. thailandensis* | E0236 | 1992 | Soil | Thailand | 345 | This study |
| 20 | 44 | *B. thailandensis* | E0260 | 1993 | Soil | Thailand | 345 | This study |
| 21 | 45 | *B. thailandensis* | E0263 | 1993 | Soil | Thailand | 345 | This study |
|  | 46 | *B. thailandensis* | E0416 | 2001 | Soil | Thailand | 345 | This study |
|  | 47 | *B. thailandensis* | E0423 | 2001 | Soil | Thailand | 345 | This study |
|  | 48 | *B. thailandensis* | E0424 | 2001 | Soil | Thailand | 345 | This study |
|  | 49 | *B. thailandensis* | E0436 | 2002 | Soil | Thailand | 345 | This study |
| 22 | 50 | *B. thailandensis* | E0438 | 2002 | Soil | Thailand | 345 | This study |
| 23 | 51 | *B. thailandensis* | E0440 | 2002 | Soil | Thailand | 345 | This study |
| 24 | 52 | *B. thailandensis* | E0048 | 1990 | Soil | Thailand | 352 | This study |
| 25 | 53 | *B. thailandensis* | E0169 | 1991 | Soil | Thailand | 352 | This study |
| 26 | 54 | *B. thailandensis* | E0174 | 1991 | Soil | Thailand | 352 | This study |
| 27 | 55 | *B. thailandensis* | E0177 | 1991 | Soil | Thailand | 352 | This study |
|  | 56 | *B. thailandensis* | E0331 | 1998 | Soil | Thailand | 352 | This study |
| 28 | 57 | *B. thailandensis* | E0188 | 1992 | Soil | Thailand | 355 | This study |
| 29 | 58 | *B. thailandensis* | E0234 | 1992 | Soil | Thailand | 355 | This study |
| 30 | 59 | *B. thailandensis* | E0336 | 1998 | Soil | Thailand | 356 | This study |
| 31 | 60 | *B. thailandensis* | E0202 | 1992 | Soil | Thailand | 357 | This study |
| 32 | 61 | *B. thailandensis* | E0367 | 1999 | Soil | Thailand | 358 | This study |
| 33 | 62 | *B. thailandensis* | E0017 | 1990 | Soil | Thailand | 359 | This study |
| 34 | 63 | *B. thailandensis* | E0131 | 1991 | Soil | Thailand | 360 | This study |
| 35 | 64 | *B. thailandensis* | E0146 | 1991 | Soil | Thailand | 360 | This study |
| 36 | 65 | *B. thailandensis* | E0159 | 1991 | Soil | Thailand | 360 | This study |
| 37 | 66 | *B. thailandensis* | E0161 | 1991 | Soil | Thailand | 360 | This study |
|  | 67 | *B. thailandensis* | E0162 | 1991 | Soil | Thailand | 360 | This study |
| 38 | 68 | *B. thailandensis* | E0153 | 1991 | Soil | Thailand | 361 | This study |
| 39 | 69 | *B. thailandensis* | E0192 | 1992 | Soil | Thailand | 361 | This study |
| 40 | 70 | *B. thailandensis* | E0253 | 1992 | Soil | Thailand | 361 | This study |
| 41 | 71 | *B. thailandensis* | E0291 | 1994 | Soil | Thailand | 361 | This study |
| 42 | 72 | *B. thailandensis* | E0305 | 1994 | Soil | Thailand | 361 | This study |
| 43 | 73 | *B. thailandensis* | E0433 | 2002 | Soil | Thailand | 361 | This study |
| 44 | 74 | *B. thailandensis* | E0274 | 1993 | Soil | Thailand | 362 | This study |
| 45 | 75 | *B. thailandensis* | E0360 | 1992 | Soil | Thailand | 362 | This study |
| 46 | 76 | *B. thailandensis* | E0257 | 1992 | Soil | Thailand | 363 | This study |
|  | 77 | *B. thailandensis* | G32 | Not known | Soil | Not known | 80 | Godoy et al. |
|  | 78 | *B. thailandensis* | E216 | 1992 | Soil | Thailand | 80 | Godoy et al. |
|  | 79 | *B. thailandensis* | E111 | 1991 | Soil | Thailand | 80 | Godoy et al. |
|  | 80 | *B. thailandensis* | E294 | 1994 | Soil | Thailand | 79 | Godoy et al. |
|  | 81 | *B. thailandensis* | E125 | 1991 | Soil | Thailand | 77 | Godoy et al. |
|  | 82 | *B. thailandensis* | LE1 | 1999 | Soil | Laos | 76 | Godoy et al. |
|  | 83 | *B. thailandensis* | VN 536b | 1997 | Soil | Vietnam | 75 | Godoy et al. |
|  | 84 | *B. thailandensis* | E27 | 1990 | Soil | Thailand | 74 | Godoy et al. |
|  | 85 | *B. thailandensis?* | 1992/2572 | 1992 | Water | Kenya | 73 | Godoy et al. |
|  | 86 | *B. thailandensis?* | 82172 | 1988 | Chicken | France | 73 | Godoy et al. |
| 47 | 87 | *B. thailandensis* | 200301589/Tx DOH | 2003 | Human infection | USA | 101 | Sequenced strain |
| 48 | 88 | *B. thailandensis* | E264 | 1993 | Soil | Thailand | 80 | Sequenced strain |
|  | 89 | *B. thailandensis* | 4 | 2007 | Soil | Australia | 699 | Sequenced strain |
|  | 90 | *B. thailandensis* | 700388 (E264) | 1994 | Soil | Thailand | 80 | Sequenced strain |
| 49 | 91 | *B. thailandensis* | CDC2721121 | 1997 | Human Wound | USA | 101 | This study |
| 50 | 92 | *B. thailandensis* | E555 | 2005 | Soil | Cambodia | 696 | This study |

**Additional data file 17. List of Bt Strains used for MLST Analysis.** This table lists 92 Bt strains used for MLST analysis, including 10 previously-reported Bt isolates (Godoy, sequence data obtained from the mlst.net database), and 4 sequenced strains where the ST (sequence type) was derived *in silico.* The 50 strains used for aCGH analysis are highlighted in light grey, and numbered for reference.
