# Supplementary material for: Genomic acquisition of a capsular polysaccharide virulence cluster by non-pathogenic Burkholderia isolates
Source: Genome Biol. 2010 Aug 27;11(8):R89. doi: 10.1186/gb-2010-11-8-r89 (PMC2945791; doi:10.1186/gb-2010-11-8-r89)
Supplement: Additional file 18 — A figure showing the correlation between aCGH similarity and locus variance. [file gb-2010-11-8-r89-S18.DOC]

**Additional data file 18.** **Relationships between MLST locus variation and aCGH genomic differences between Bt strains.**

**Additional data file 18.** **Relationships between MLST locus variation and aCGH genomic differences between Bt strains.**

Top : Strains were clustered according their overall similarities in aCGH profiles (see Main Text).

Middle : Beginning with pairs of highly-correlated strains (lowest levels on clustering tree), we computed the degree of MLST locus variance (based on the 7 MLST housekeeping genes) for each strain pair. The locus variance rates for each pair is displayed along the row designated “Locus Variant”. The maximum degree of locus variance observed in the data set is 4.

Bottom : aCGH genomic differences between strains are reflected from low (green, indicating highly similar strains) to high (orange, indicating larger aCGH differences between strains).

In general, strains showing high aCGH/genomic similarity (green, low peaks) are associated with low levels of locus variance (0-1). Conversely, strains showing low aCGH/genomic similarity (orange, high peaks) tend to show higher locus variance (3-4).
