# Supplementary material for: Genomic acquisition of a capsular polysaccharide virulence cluster by non-pathogenic Burkholderia isolates
Source: Genome Biol. 2010 Aug 27;11(8):R89. doi: 10.1186/gb-2010-11-8-r89 (PMC2945791; doi:10.1186/gb-2010-11-8-r89)
Supplement: Additional file 19 — A rooted phylogenetic tree (MLST) confirming that the variant strains are distinct from the rest of the Bt strains. [file gb-2010-11-8-r89-S19.DOC]

**Additional data file 19. MLST segregation of Bt into two clades**


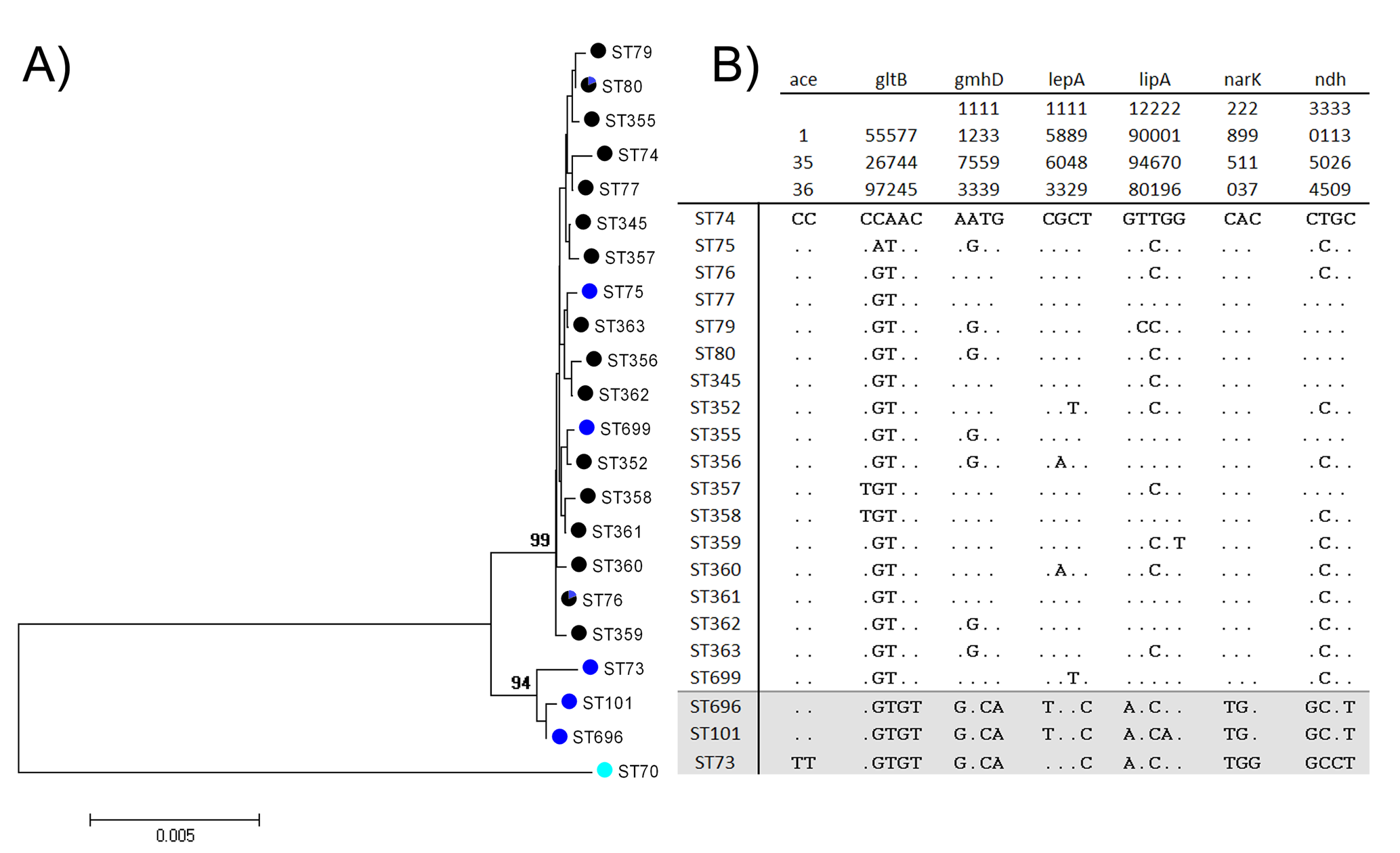


**Additional data file 19. MLST segregation of Bt into two clades**

1. Neighbor-joining tree constructed from the concatenated sequences of the seven MLST loci of *Burkholderia*. A total of 22 concatenated sequences obtained from 21 STs of *B. thailandensis* and one ST of *B. pseudomallei* were used to construct a neighbor-joining tree. The light blue dot represents *B. pseudomallei* ST70. Black dots represent *B. thailandensis* found only in Thailand. Dark blue dots represent *B. thailandensis* found outside of Thailand, including ST75 (1 strain, Vietnam), ST76 (1 strain, Laos), ST80 (1 strain out of 19, unknown, from Godoy’s study), ST699 (1 strain, Australia), ST73 (2 strains, France and Kenya), ST696 (Cambodia), and ST101 (2 strains, USA). The percent recoveries of the major nodes in 1,000 bootstrap replicates are shown. The bar indicates differences at 0.5% of nucleotide sites.
2. Polymorphic Sites in Seven MLST Housekeeping Gene Fragments of 21 Bt Sequence Types. The nucleotides present at each variable site for the 21 STs compared with those present in ST74 (allelic profile 6-10-15-8-10-14-9). For the other alleles, only those sites that differ are shown; those that are the same as in the alleles of ST74 are shown by periods. Nucleotide sites that are the same in all alleles are not shown. The position of each polymorphic site within the sequenced fragment is shown above, in vertical format. The STs highlighted in grey belong to the clade containing Bt strains with Bp-likeCPS. STs in this cluster showed more genetic variability than the remaining STs which all reside in a second, larger clade.
