# Supplementary material for: Genomic acquisition of a capsular polysaccharide virulence cluster by non-pathogenic Burkholderia isolates
Source: Genome Biol. 2010 Aug 27;11(8):R89. doi: 10.1186/gb-2010-11-8-r89 (PMC2945791; doi:10.1186/gb-2010-11-8-r89)
Supplement: Additional file 20 — A graph that charts the growth rate of the reference strain BtE264, BtE555 and the mutant CPS KO in rich media. [file gb-2010-11-8-r89-S20.DOC]

**Additional data file 20. Growth rates of Bt strains.**


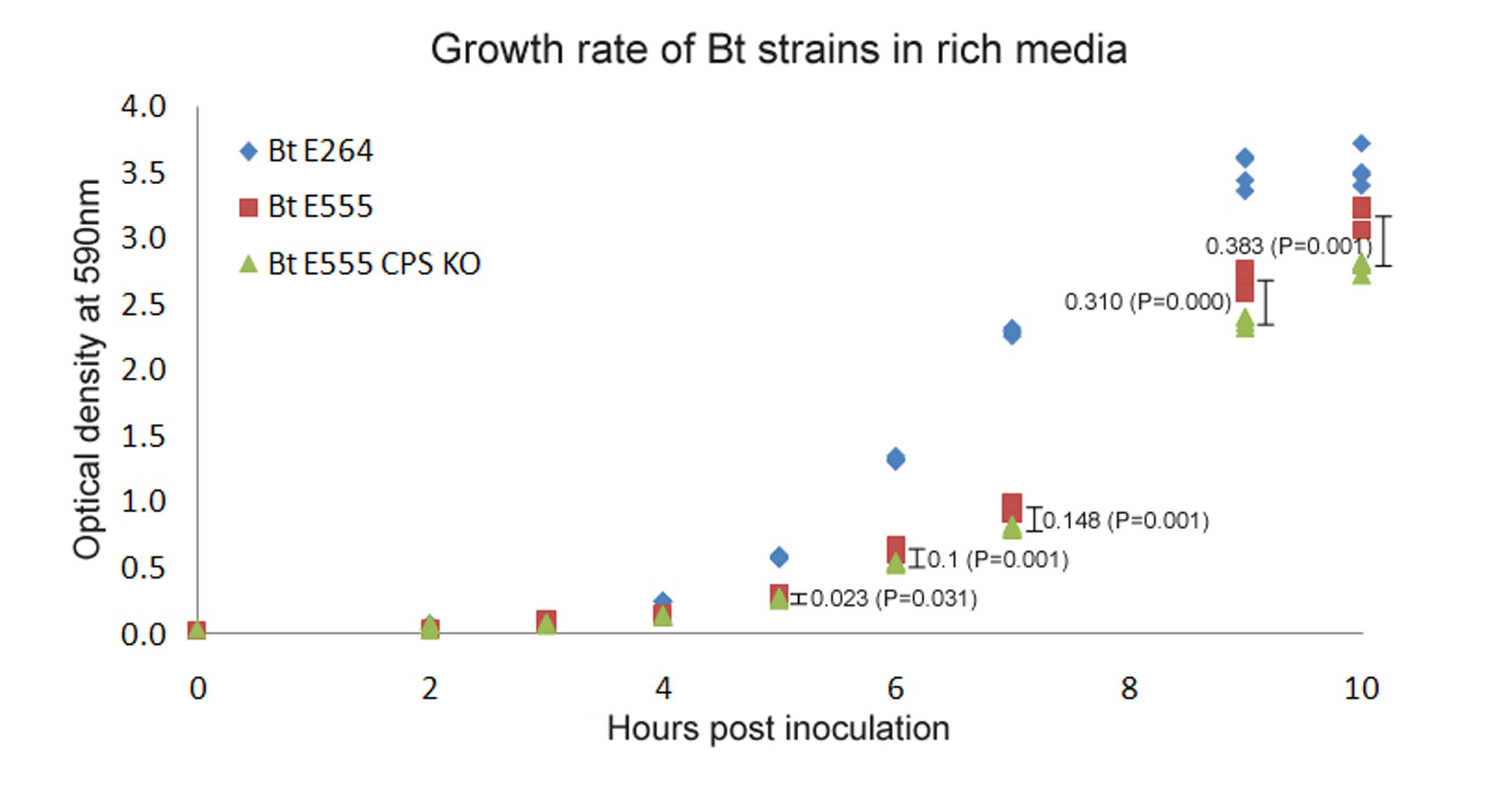


**Additional data file 20. Growth rates of Bt strains.**

In growth experiments, BtE555 CPS KO strains grew at slightly slower rates than BtE555 wild-type strains, but these differences while significant were marginal (note the small differences between the averages shown, next to the respective P values). The growth curve of the BtE264 reference strain is also shown.
